# Enhanced removal of phenol red from water by Cu(ii)-modified Mg–Al composites *via* adsorption and photocatalysis

**DOI:** 10.1039/d5ra02645h

**Published:** 2025-08-11

**Authors:** Van Nhuong Vu, Thi Ha Thanh Pham, Thi Huong Le, Truong Xuan Vuong

**Affiliations:** a Faculty of Chemistry, Thai Nguyen University of Education No. 20 Luong Ngoc Quyen Street Thai Nguyen City 24000 Vietnam; b Faculty of Natural Sciences and Technology, TNU-University of Science Tan Thinh Ward Thai Nguyen City 24000 Vietnam xuanvt@tnus.edu.vn

## Abstract

This study investigates Cu(ii)-modified Mg–Al layered double hydroxide (LDH) composites for efficient removal of phenol red (PR) from water through adsorption and visible-light photocatalysis. The aim was to develop a sustainable material capable of addressing dye pollution in textile wastewater. The composites were synthesized *via* co-precipitation with optional calcination, non-calcined and calcined materials. Structural characterization (XRD, EDX, FT-IR, SEM) confirmed successful Cu^2+^ incorporation by isomorphic substitution, forming mesoporous, less crystalline structures. Uncalcined samples, particularly 5-CuH, retained a layered morphology and removed up to 94% of PR. The removal occurred *via* electrostatic attraction, π–π interactions, and ion exchange. Cu^2+^ narrowed the bandgap (*E*_g_ ≈ 2.1–3.1 eV), enhancing photocatalytic activity. Under visible light, 5-CuH degraded approximately 40% of PR within 60 minutes. Calcined samples (*e.g.*, 5-CuH500) formed CuO particles and performed better under acidic conditions despite reduced structural integrity. Both materials reduced COD by over 90% in real textile wastewater, confirming their dual-function performance, adsorption and photocatalysis. In conclusion, Cu–Mg–Al composites offer an effective, eco-friendly alternative for treating dye-contaminated wastewater.

## Abstract 2

This study investigates Cu(ii)-modified Mg–Al layered double hydroxide (LDH) composites for efficient removal of phenol red (PR) from water through adsorption and visible-light photocatalysis.

## Graphical abstract

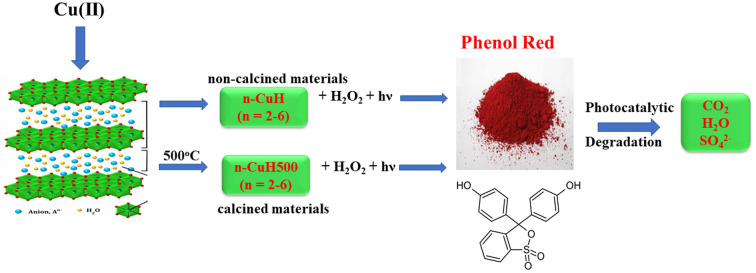

## 1. Introduction

Synthetic dyes like phenol red (PR) are extensively used in textile, food processing, and pharmaceutical industries.^1,2^ Although employed for production, such dyes are recalcitrant and toxic, and therefore inflict immense damage on aquatic organisms upon being released into water bodies.^3^ phenol red, commonly employed as a pH indicator,^3^ is not easily biodegradable and can be dangerous to aquatic organisms and human beings.^4^ Its harmful impacts have been documented by research, including respiratory dysfunction and organ injury in aquatic life.^2^

The environmental impact does not end with ecosystems. PR accumulated in water has also been linked with severe health risks such as cancer, genetic impairment, and disease of the reproductive system.^5^ Thus, the treatment of colored wastewater is required to safeguard not only environmental health but also public health.

Of the available treatments, adsorption is in general reputed to be easy to apply, low-cost, and very effective at removal, even at low concentrations of pollutants.^6^ Activated carbon, clays, and layered double hydroxides (LDHs) are typical adsorbents.^7–9^ However, issues still remain regarding optimizing material efficiency without sacrificing economic scalability.^10^ Activated carbon and clays are widely used adsorbents but each has notable limitations. Activated carbon, while effective, is costly to produce, difficult to regenerate, and lacks selectivity.^11,12^ Clays typically have low adsorption capacity, slow kinetics, and are sensitive to pH changes, often aggregating in water.^13^

Photocatalytic degradation, another effective process, uses light to activate catalysts that generate reactive oxygen species (ROS), which break down organic dyes.^14^ The process is very efficient under sunlight.^15^ Weak visible-light absorption, however, is a drawback that reduces the efficiency of most photocatalysts.^16^ Therefore, scientists have looked for advanced materials, including heterojunctions and polyoxometalate-based systems, to increase efficiency and broaden applicability.^17,18^

To overcome the limitations of a single method, a hybrid process integrating adsorption and photocatalysis in an integrated material system has been achieved. The approach utilizes the pre-concentration of pollutants through adsorption to enhance the efficiency of photocatalytic degradation.^19^ LDHs, particularly Zn–Al and Mg–Al types, are highly promising candidates due to their adjustable structures, high surface areas, and multi-functionality.^9,20,21^

Recent studies have shown that doping Zn–Al LDHs with transition metal ions like Cu^2+^ greatly increases photocatalytic activity, primarily by enhancing charge separation and improving visible-light response.^9^ Doping by Cu^2+^ also augments adsorption, ensuring dual-functionality for dye cleaning.^22^ Synergistic interaction between photocatalysis and adsorption creates more active sites and facilitates efficient degradation channels. Cu^2+^-modified Mg–Al layered double hydroxides (LDHs) have been synthesized and utilized as efficient catalysts for the synthesis of organic compounds,^23–25^ and the removal of methyl orange from water^23^ owing to their unique structural and physicochemical properties. Despite such advancements, the application of Cu^2+^-modified Mg–Al LDHs towards the removal of phenol red has not been adequately explored. The mechanism behind Cu^2+^-promoted photocatalysis is not yet fully understood.^9,26^ To address this knowledge gap, the present work develops a Cu^2+^-modified Mg–Al LDH system that integrates adsorption and photocatalysis to realize the effective and sustainable removal of phenol red from wastewater.

The present study focuses on the synthesis and evaluation of Cu^2+^-modified Mg–Al layered double hydroxides (LDHs) for the effective removal of phenol red (PR) from aqueous solutions. The proposed Cu(ii)-modified LDH composite is designed to serve as a multifunctional material with several desirable properties: (i) high adsorption capacity for PR due to its well-ordered layered structure; (ii) the ability to initiate photocatalytic degradation of PR under visible light irradiation; (iii) strong structural stability, enabling repeated use without significant performance loss; and (iv) efficient operation under near-neutral pH conditions, enhancing its applicability in practical water treatment systems. To the best of our knowledge, this is the first report on the synthesis of a Cu(ii)-modified Mg–Al LDH composite specifically tailored for the efficient removal of phenol red from water.

This research pursues three main objectives: (1) to investigate the adsorption performance of the modified LDHs; (2) to evaluate their photocatalytic activity under visible light; and (3) to examine the synergistic effect of combining adsorption and photocatalysis.

We hypothesize that Cu^2+^ modification enhances the material's performance through increased surface area, improved charge separation, and the inherent catalytic activity of Cu^2+^ under light activation. By integrating adsorption and photocatalysis into a single system, this approach offers a more efficient and sustainable method for the removal of phenol red from water.

## 2. Materials and methods

### 2.1. Materials

#### 2.1.1. Reagents and chemicals used

The chemicals used in this study include: Mg(NO_3_)_2_·6H_2_O, Al(NO_3_)_3_·9H_2_O, Cu(NO_3_)_2_·3H_2_O, Na_2_CO_3_, phenol red, and K_2_Cr_2_O_7_. All chemicals were of analytical grade and obtained from Merck, Germany. These chemicals were used without the addition of any further additives, ensuring high purity for the experiments.

### 2.2. Synthesis of Cu-modified Mg–Al materials

#### 2.2.1. Detailed steps for the co-precipitation method

The chemicals Al(NO_3_)_3_·9H_2_O, Mg(NO_3_)_2_·6H_2_O, and Cu(NO_3_)_2_·3H_2_O (in specific molar ratios) were dissolved in 150 mL of distilled water to obtain solution A. The reaction vessel containing solution A was placed on a magnetic stirrer and maintained at room temperature, stirred for 30 minutes at a speed of 500 rpm. Subsequently, 25 mL of 0.6 M Na_2_CO_3_ was added slowly, and stirring was continued for an additional 30 minutes. After 30 minutes of stirring, the gel was aged in a 500 mL glass beaker at 70 °C for 24 hours. Following gel aging, the product was washed several times with hot water (at 70 °C) until the pH reached 7.0. The resulting solid was then dried at 80 °C for 24 hours to obtain hydrotalcite MgAlCO_3_ (denoted as H) and Cu^2+^-modified hydrotalcite (denoted as *n*-CuH, where *n* represents the molar ratio of Cu^2+^ in the sample).^26^

The hydrotalcite and Cu-hydrotalcite samples were subsequently calcined at 500 °C for 5 hours with a heating rate of 3 °C min^−1^, and were labeled as H500 and *n*-CuH500, respectively. A detailed description of the sample nomenclature is provided in Table 1.

**Table 1:** Molar ratios of Cu, Mg, Al, and CO_3_ in different Cu^2+^-modified Mg–Al samples

Sample	Molar ratio of elements Cu : Mg : Al : CO_3_
H (H500)	0 : 0.07 : 0.03 : 0.015 (0 : 7:3 : 1.5)
2-CuH (2-CuH500)	0.02 : 0.05 : 0.03 : 0.015 (2 : 5:3 : 1.5)
3-CuH (3-CuH500)	0.03 : 0.04 : 0.03 : 0.015 (3 : 4:3 : 1.5)
4-CuH (4-CuH500)	0.04 : 0.03 : 0.03 : 0.015 (4 : 3:3 : 1.5)
5-CuH (5-CuH500)	0.05 : 0.02 : 0.03 : 0.015 (5 : 2:3 : 1.5)
6-CuH (6-CuH500)	0.06 : 0.01 : 0.03 : 0.015 (6 : 1:3 : 1.5)

The samples were then ground using a mortar and pestle and were employed for the characterization of their structural properties and photocatalytic activity in the degradation of phenol red and dye pollutants in textile wastewater.

#### 2.2.2. Characterization techniques

##### 2.2.2.1. X-ray diffraction (XRD)

The XRD measurement method provides detailed insights into the crystal structure and properties of the studied material. X-ray diffraction was performed using a D8-Advance 5005 diffractometer. The measurement conditions were as follows: Cu Kα radiation (*λ* = 1.5406 Å), a temperature of 25 °C, a 2*θ* scanning range from 5° to 70°, and a scanning speed of 0.03° s^−1^.

##### 2.2.2.2. Fourier transform infrared (FTIR) spectroscopy

FT-IR is an essential tool for investigating the structural characteristics and changes of Cu/Mg/Al materials, providing a deeper understanding of their mechanisms of action and potential applications in photocatalysis and pollution treatment studies. The FT-IR spectra were recorded in the wavelength range from 4000 cm^−1^ to 400 cm^−1^.

##### 2.2.2.3. Scanning electron microscopy (SEM)

The morphology of the studied material was characterized using a scanning electron microscope (SEM) on a Hitachi S-4800 instrument.

##### 2.2.2.4. Energy dispersive X-ray spectroscopy (EDX)

EDX was used to analyze the elemental composition and distribution of elements within the studied material. EDX helps to identify the presence and percentage of elements on the material's surface, thus aiding in the evaluation of its structure and properties. The chemical composition of the elements was determined through EDX analysis on a HORIBA instrument (MODEL 7593-H).

##### 2.2.2.5. Nitrogen adsorption and desorption isotherms (BET)

Nitrogen adsorption and desorption isotherms (BET) were used to determine the specific surface area, pore size distribution, and porous structure of the studied material. This method helps evaluate the material's adsorption capacity, providing important information about its structure and surface properties, which influence its performance in various applications. The N_2_ adsorption and desorption isotherms (BET) were conducted using a TriStar II Plus 2.03 instrument.

##### 2.2.2.6. UV-Vis diffuse reflectance spectroscopy (UV-Vis DRS)

UV-Vis DRS is a valuable tool for analyzing the optical properties, electronic structure, and photocatalytic efficiency of the studied material, particularly in pollution treatment and photocatalysis applications. The UV-Vis diffuse reflectance spectra of the solid samples were recorded using a U-4100 Spectrophotometer.

### 2.3. Adsorption studies

A 250 mL solution of phenol red (concentration 100 ppm) and 0.2 g of the material were placed in a 500 mL beaker, which was sealed with a black plastic bag. The mixture was then stirred on a magnetic stirrer at room temperature. Samples were taken at various time intervals (30, 60, 90, 120, 150, 180 minutes) for analysis. Afterward, the phenol red solution was centrifuged, and the absorbance was measured using a UV-Vis 1700 spectrophotometer. The adsorption efficiency of phenol red by the material was calculated using the following formula.^9^1
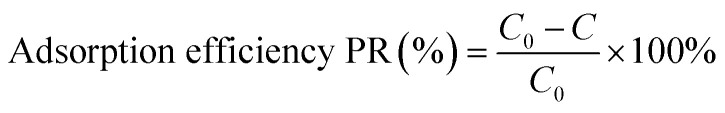


where: *C*_0_ is the initial concentration of phenol red, *C* is the concentration of phenol red at the time of sampling.

The adsorption capacity of 12 synthesized material samples (6 non-calcined and 6 calcined at 500 °C) was investigated. All experiments were repeated three times. From the results, the adsorption efficiency and the equilibrium adsorption time for the materials toward phenol red in aqueous solution were determined.

### 2.4. Photocatalytic degradation studies

#### 2.4.1. Effect of illumination time

A 250 mL solution of phenol red (concentration 100 mg L^−1^) and 0.2 g of the material were placed in a 500 mL beaker, which was sealed with a black plastic bag. The mixture was then stirred on a magnetic stirrer for 150 minutes at room temperature to achieve adsorption equilibrium of the material. Samples were filtered for adsorption efficiency determination. Subsequently, 1.2 mL of 30% H_2_O_2_ was added to the beaker. The beaker was placed on a magnetic stirrer (at 500 rpm) and illuminated with a 30 W LED light source.^9^

To assess the phenol red degradation capacity of the material series over time, samples were taken at fixed intervals (every 30 minutes). Afterward, the samples were centrifuged, diluted five times, and analyzed using a UV-Vis 1700 spectrophotometer. From the obtained results, the photocatalytic degradation efficiency was determined using the following eqn (2)2
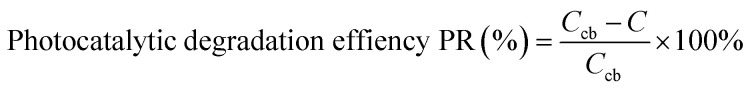


where: *C*_cb_: concentration of phenol red (PR) at adsorption equilibrium, *C*: concentration of phenol red (PR) at the time of sampling.

However, both adsorption and photocatalytic degradation processes conducted simultaneously during the treatment of phenol red. Therefore, the degradation efficiency of phenol red can be determined according to the eqn (3):

3 Photocatalytic degradation efficiency PR (%) = treatment efficiency PR (%) − adsorption efficiency PR at adsorption equilibrium (%)

The treatment efficiency of phenol red (including adsorption and photocatalytic degradation) was determined according to eqn (4):4
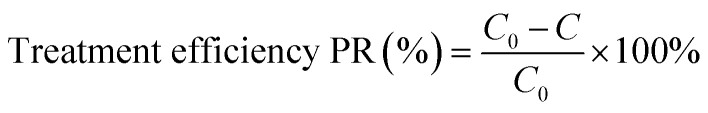


where: *C*_0_ is the initial concentration of phenol red, *C* is the concentration of phenol red at the time of sampling.

The degradation capacity of the 12 synthesized material samples was investigated. All experiments were repeated three times. From the obtained results, the phenol red degradation efficiency of the synthesized material samples over time and in relation to the Cu^2+^ ion ratio in the materials was evaluated.

#### 2.4.2. Effect of Cu^2+^ ion ratio in non-calcined and calcined samples

From the results of the investigation on the effect of illumination time on phenol red degradation, we also obtained data reflecting the phenol red degradation efficiency as a function of the molar ratio of Cu^2+^ ions in both non-calcined material samples and those calcined at 500 °C. Additionally, we were able to compare the catalytic activity of the material samples to identify the most active catalysts for further studies, as well as to compare the catalytic activity between the non-calcined and calcined samples at 500 °C.^22^

#### 2.4.3. Effect of phenol red concentration in aqueous solution

Based on the results obtained above, two material samples with the best catalytic activity for investigating the effect of pH on phenol red degradation under LED light (one non-calcined sample and one calcined sample) were selected. The experiment with 250 mL of phenol red at different concentrations (100, 125, 150, 175, and 200 ppm) and 0.2 g of the optimal catalyst, using a 30 W LED light source at room temperature were conducted.

The experimental procedure was carried out in the same manner as described above, simultaneously for all five concentrations of phenol red (PR). All experiments were repeated three times. From the obtained results, we were able to assess the effect of PR concentration on the catalytic activity of the material.^9^

#### 2.4.4. Effect of pH

Two material samples with the best catalytic activity were selected to investigate the effect of environmental pH on phenol red degradation under LED light. The experiment was conducted with 250 mL of phenol red at a concentration of 150 ppm and 0.2 g of the non-calcined material sample; and phenol red at 100 ppm with 0.2 g of the calcined material sample at 500 °C. The pH values of the solutions were adjusted using 0.1 M NaOH and 0.1 M HCl to achieve the desired pH values of 2, 2.5, 3, 3.5, 6, 8, and 10.^9^ The samples, with adjusted pH, were treated simultaneously following the procedure described above. This allowed the determination of phenol red degradation efficiency over time when investigating the effect of environmental pH. From the obtained results, the optimal pH range for phenol red degradation was selected.

### 2.5. Investigation of the dyeing wastewater treatment capacity of the material

The wastewater was collected from the dyeing wastewater tank in a traditional craft village. The wastewater appeared dark red in color. Due to the high concentration of phenol red in the initial samples, they were diluted 10 times for investigating the degradation capacity of the synthesized material samples.

A 250 mL diluted wastewater sample was placed in a 500 mL beaker, and the pH of the solution was adjusted to different values within the optimal pH range. Then, 0.2 g of the optimal material sample was added, and the beaker was sealed with a black plastic bag. The mixture was stirred on a magnetic stirrer for 150 minutes at room temperature to achieve adsorption equilibrium of the material. Subsequently, 5 mL of 30% H_2_O_2_ was added to the beaker, which was again sealed with the black plastic bag and illuminated under a 30 W LED light source at room temperature.^9^ Samples were taken every 60 minutes to determine the degradation of colorants and the mineralization of organic compounds in the wastewater for both non-calcined and calcined synthesized material samples.^9^

The degradation efficiency of colorants in the wastewater sample is determined using the following formula: 5
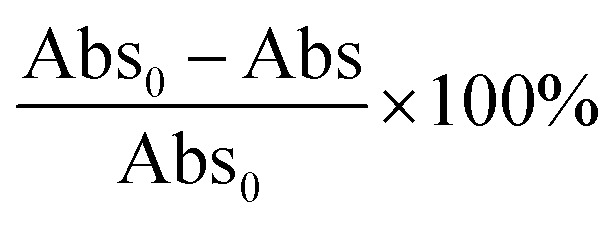
where, Abs_0_ is the absorbance of the colorant at the maximum wavelength at the initial time; Abs is the absorbance of the colorant at the maximum wavelength at the time of sampling.

The mineralization efficiency of COD in the wastewater is determined using the following formula: 6
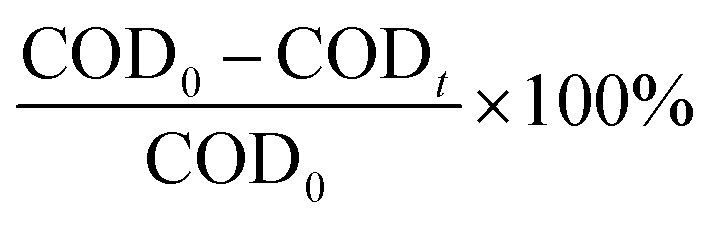


Chemical Oxygen Demand (COD) is determined using the K_2_Cr_2_O_7_ method, with detailed procedures provided in the previous studies.^9^

## 3. Results and discussion

### 3.1. Characterization of Cu^2+^-Modified Mg–Al materials

#### 3.1.1. X-ray diffraction patterns (XRD) of materials

**Fig. 1:**
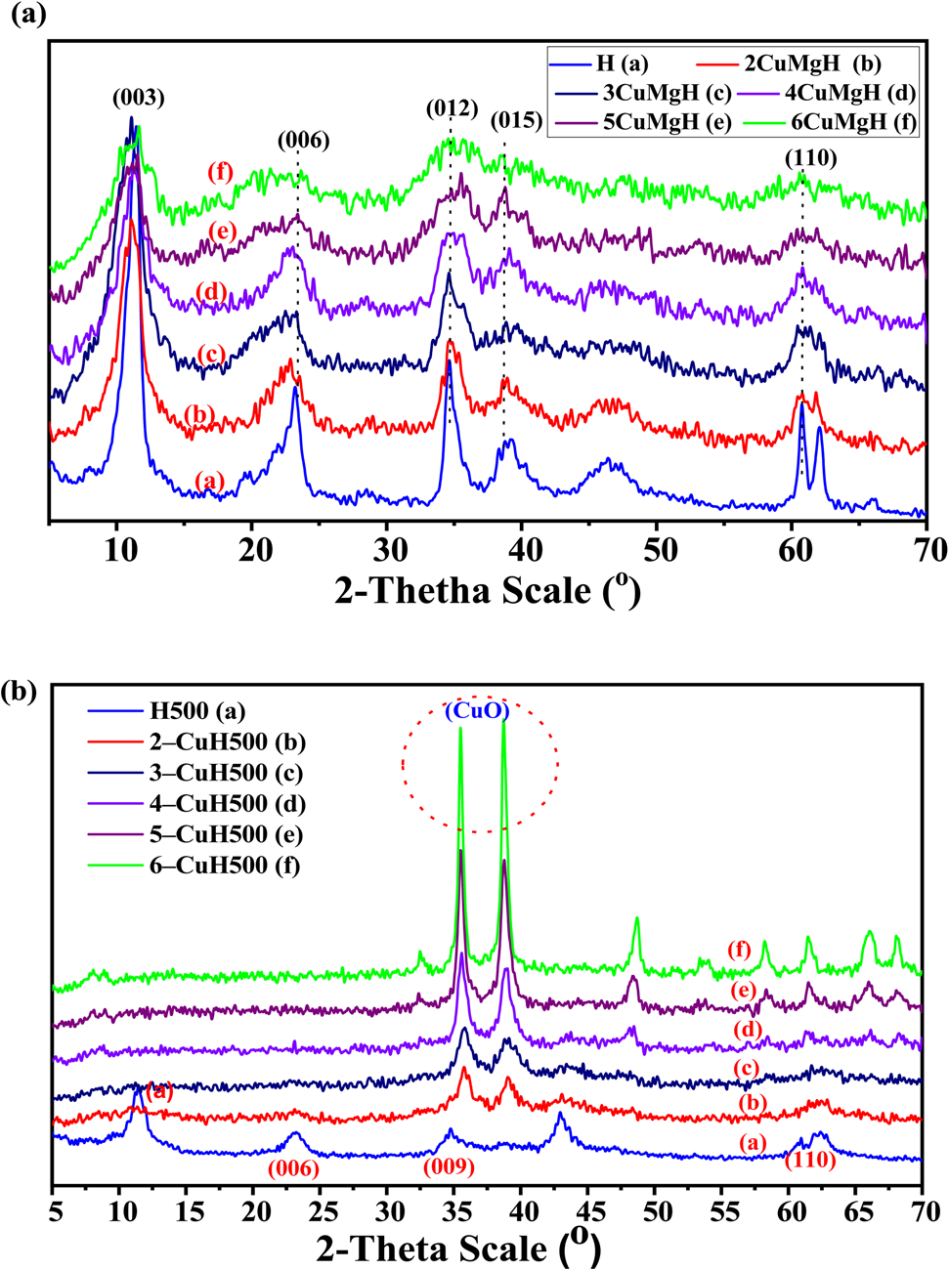
XRD patterns of the non-calcined (a) and calcined samples (b).

Fig. 1a shows the XRD patterns of the uncalcined CuMgAlCO_3_ material samples. All samples exhibit characteristic diffraction peaks indicative of a hydrotalcite-like crystalline structure, with most diffraction peaks appearing at 2*θ* angles of 11.57° (*d*_003_), 23.45° (*d*_006_), and 60.9° (*d*_110_), among others. However, the intensity of these peaks, particularly at 2*θ* = 11.57° (*d*_003_), significantly decreases with increasing Cu^2+^ content in the materials. This suggests degradation of the hydrotalcite-like layered structure upon Cu^2+^ modification of MgAlCO_3_ hydrotalcite materials.^9,22^

Table 2 illustrates the *d*_003_ values, representing the interlayer spacing. For uncalcined MgAlCO_3_ and CuMgAlCO_3_ samples, *d*_003_ values range from 7.66 to 8.03 Å, which is characteristic of hydrotalcite-like structures and confirms the presence of interlayer CO_3_^2−^ anions.^27^ Additionally, the weakening of the characteristic hydrotalcite diffraction peaks can be attributed to the isomorphic substitution of Mg^2+^ by Cu^2+^ ions and the slightly larger ionic radius of Cu^2+^ (0.073 nm) compared to that of Mg^2+^ (0.072 nm).^24^ Although some of the uncalcined samples appear black in colour, no diffraction peaks corresponding to crystalline CuO, such as the one at 2*θ* = 35.4° (*d*_200_), are observed.^24^ This indicates that Cu^2+^ ions are well-dispersed within the brucite-like layers of the MgAlCO_3_ hydrotalcite structure.

**Table 2:** Some characteristics of the H and *n*-CuH material samples before and after calcination^a^

Sample	*d*_003_ (Å)	*d*_006_(Å)	*d*_110_(Å)	Average crystallite size (Scherrer method) (nm)	Lattice parameters (Å)		Color
					*a*	*c*	
H	7.870	3.819	1.524	7.02	3.048	23.601	White
2-CuH	8.030	3.887	1.525	4.58	3.050	24.090	Black
3-CuH	7.843	3.852	1.521	4.17	3.042	23.880	Black
4-CuH	7.843	3.826	1.522	4.20	3.044	23.529	Black
5-CuH	7.807	3.809	1.521	3.43	3.042	23.529	Blue + black
6-CuH	7.664	3.869	1.52	3.08	3.040	23.421	Blue
H500	7.704	3.816	1.519	6.96	3.038	22.992	White
2-CuH500	—	—	—	10.24	—	—	Black
3-CuH500	—	—	—	9.27	—	—	Black
4-CuH500	—	—	—	13.49	—	—	Black
5-CuH500	—	—	—	19.56	—	—	Black
6-CuH500	—	—	—	25.49	—	—	Black

a “—”: undetermined.

Table 2 presents the calculated values of the lattice parameters *a* and *c*. The parameter a represents the distance between cations within the brucite-like layer and is calculated using the formula *a* = 2·*d*_110_. The parameter *c* corresponds to the thickness of the brucite layer combined with the interlayer spacing and is determined by *c* = 3·*d*_003_.^22,28^ The a values range from 3.04 to 3.05 Å, while the *c* values lie between 23.0 and 24.09 Å (Table 2). These parameters vary only slightly with the increase of Cu^2+^ content in the modified materials. These results indicated the preservation of the hydrotalcite-like layered structure and the isomorphic substitution of Mg^2+^ by Cu^2+^ ions within the brucite-like lattice.

The XRD analysis results of Cu^2+^-modified hydrotalcite materials are shown in Fig. 1b. After calcination at 500 °C for 5 hours, the synthesized samples exhibit significant changes in both characteristic diffraction peaks and crystalline phase composition. Diffraction peaks corresponding to the hydrotalcite structure are still observed in the H500 sample after calcination, appearing at angles associated with the (003), (006), (009), and (110) planes. This indicates that the H500 sample retains its hydrotalcite-like layered structure post-calcination, although the peak intensities are significantly reduced.

In contrast, the characteristic peaks of the hydrotalcite structure completely disappear in the Cu^2+^-modified samples after calcination. This is due to the complete decomposition of the materials into oxides such as MgO, Al_2_O_3_, and CuO.^29,30^ However, no diffraction peaks corresponding to the MgO phase are observed. Only two prominent peaks of the CuO phase appear at 2*θ* angles of 35.4° and 38.6°, with their intensities increasing progressively with the rising Cu/Al molar ratio in the samples.

The average crystallite sizes of CuO are provided in Table 2. The CuO crystallites are in the nanometer range, with sizes ranging from 9.3 to 25.5 nm. The increase in CuO crystallite size may be attributed to the aggregation of smaller particles, likely caused by the increased Cu^2+^ content in the synthesized materials.

#### 3.1.2. EDX spectral analysis results of the studied materials

**Fig. 2:**
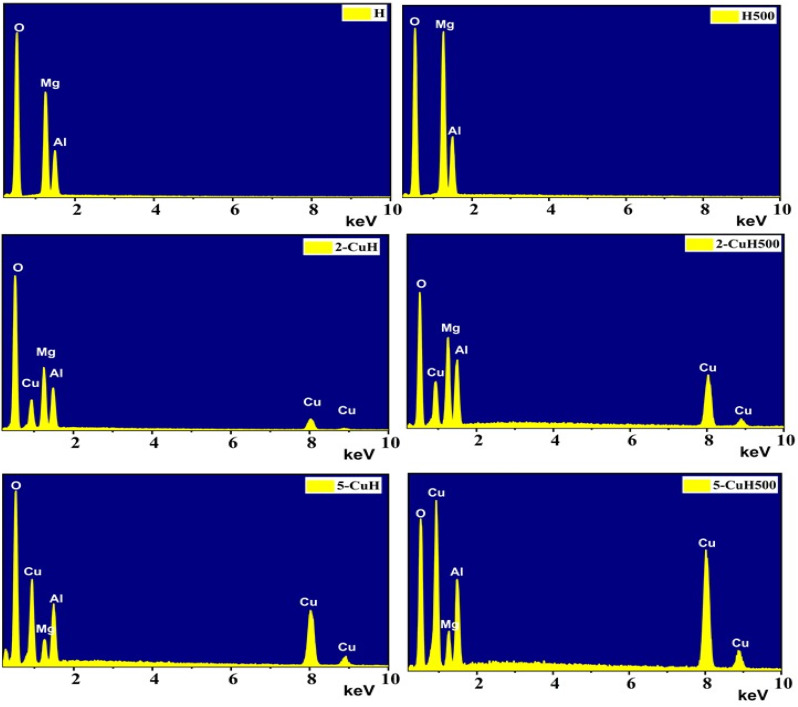
EDX spectra of the six samples: H, H500, 2-CuH, 2-CuH500, 5-CuH, and 5-CuH500.

The incorporation of metal ions into the hydrotalcite structure was further confirmed by EDX analysis. The elemental composition and atomic percentages of the materials are presented in Fig. 2 and Table 3.

**Table 3:** Elemental composition of selected elements in the synthesized materials (%)

Sample	Atomic composition of elements in studied samples (%)			
	% Mg	% Cu	% Al	% O
H	17.25	0	7.57	75.18
H500	21.35	0	8.88	69.77
2-CuH	14.15	5.14	8.11	72.60
2-CuH500	17.00	6.67	9.93	66.40
5-CuH	5.76	14.74	8.82	70.68
5-CuH500	7.97	19.82	11.18	61.02

The results indicate that all six samples, including three uncalcined (H, 2-CuH, 5-CuH) and three calcined (H500, 2-CuH500, 5-CuH500), contain the elements Mg, Cu, Al, and O. The atomic ratios of elements such as Mg : Al and Cu : Mg : Al in the analyzed samples closely match the theoretical ratios calculated for their synthesis. For example, sample H shows a Mg : Al ratio of 17.25 : 7.57 ≈ 2.28 : 1, which corresponds well with the theoretical ratio of 7 : 3. Sample 5-CuH exhibits a Cu : Mg : Al ratio of 14.74 : 5.76 : 8.82 ≈ 5.12 : 2 : 3.06, approximating the designed ratio of 5 : 2 : 3.

These EDX results confirm that the metal salt precursors were almost completely precipitated as hydroxides, which then formed the hydrotalcite and Cu^2+^-modified hydrotalcite structures.

#### 3.1.3. FT-IR analysis of studied materials

**Fig. 3:**
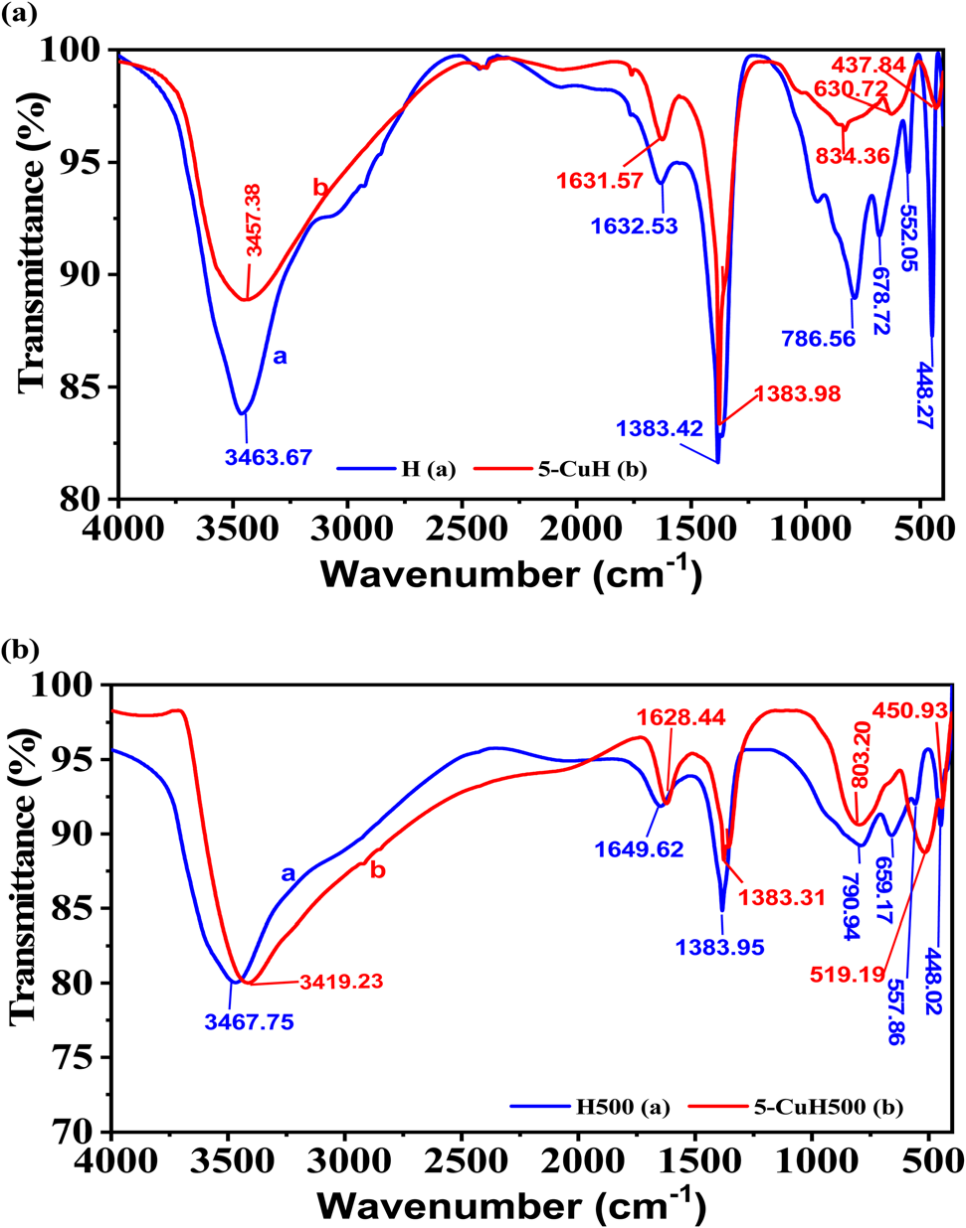
FT-IR spectra of four material samples: H and 5-CuH (a); H500 and 5-CuH500 (b).

The FT-IR spectra of four representative samples, including H and 5-CuH (Fig. 3a), H500 and 5-CuH500 (Fig. 3b), reveal several characteristic absorption bands. All four materials display a broad band in the range of 3419.23–3467.75 cm^−1^, which corresponds to the stretching vibrations of hydroxyl groups (–OH) and interlayer water molecules. The band observed around 1628.44–1649.62 cm^−1^ is attributed to the bending vibrations of both –OH groups and interlayer water.

The sharp bands at 1383.31–1383.98 cm^−1^ are characteristic of the stretching vibrations of interlayer carbonate (CO_3_^2−^) anions. In the lower wavenumber region (448.02–834.36 cm^−1^), the observed bands are associated with metal–oxygen (M–O), metal–hydroxyl (M–OH), and bridging oxygen vibrations (O–M–O, M–O–M), indicating the presence of Cu^2+^, Mg^2+^, and Al^3+^ cations within the brucite-like layers. Both 5-CuH and 5-CuH500 samples exhibit additional peaks at 834.36 and 803.20 cm^−1^, which are assigned to Cu^2+^ species incorporated into the hydrotalcite and LDH structures. These results confirm the presence of interlayer carbonate anions and the successful incorporation of Cu^2+^ ions into the brucite-like lattice of the synthesized materials.^9,31^

#### 3.1.4. SEM analysis of studied materials

**Fig. 4:**
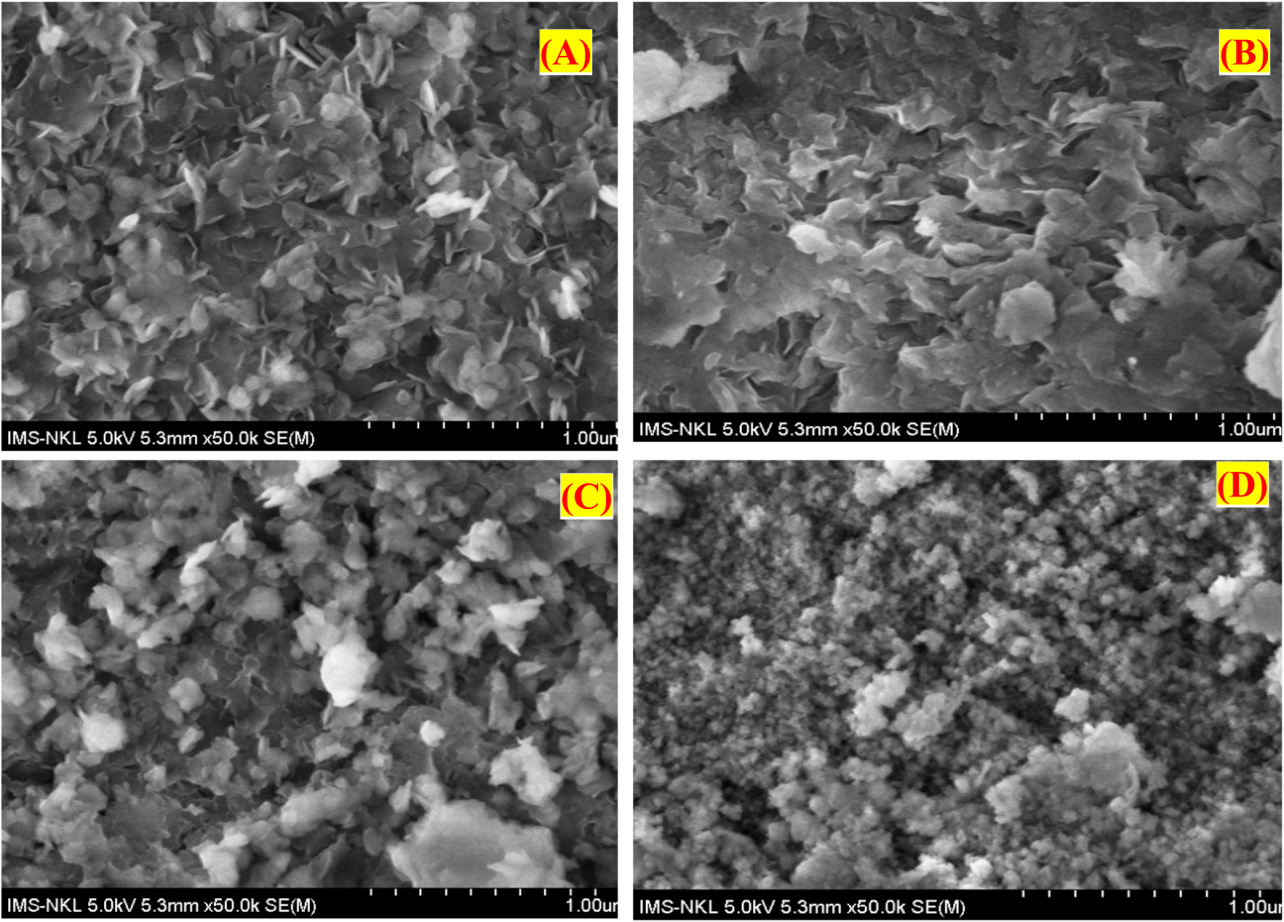
SEM images of the synthesized material samples (H (A), 5-CuH (B), H500 (C), 5-CuH500 (D)). (H: MgAlCO_3_ hydrotalcite, dried at 80 °C for 24 hours; H500: H sample calcined at 500 °C for 5 hours; 5-CuH: Cu^2+^-modified hydrotalcite with a Cu^2+^/Mg^2+^ molar ratio of 5%, dried at 80 °C for 24 hours; 5-CuH500: 5-CuH sample calcined at 500 °C for 5 hours).

Fig. 4 presents the SEM images of four synthesized material samples: H, H500, 5-CuH, and 5-CuH500. The results show that the samples H, H500, and 5-CuH exhibit the characteristic plate-like morphology of hydrotalcite structures. Among them, sample H displays more uniform and well-defined nanosheets, with interlaced platelets ranging from several tens to approximately 100 nm in size. In contrast, the 5-CuH sample shows larger platelets, which is likely due to the aggregation of smaller sheets into larger structures.

After calcination, the H500 samples had structural changes due to the decomposition of hydroxide groups and interlayer carbonates. Although the layered hydrotalcite-like structure was partially retained, many sheets were broken into smaller fragments or aggregated into larger ones, resulting in less uniform morphology compared to the uncalcined H sample.

In contrast, the 5-CuH500 sample exhibited the destruction of the hydrotalcite-like layered structure. The SEM image of 5-CuH500 revealed the presence of small nanoparticles (tens of nanometers) and larger aggregates (>100 nm), with no visible layered morphology. These SEM observations were in good agreement with the XRD analysis results, confirming the structural changes induced by Cu^2+^ incorporation and thermal treatment.

#### 3.1.5. BET analysis of the studied materials

**Fig. 5:**
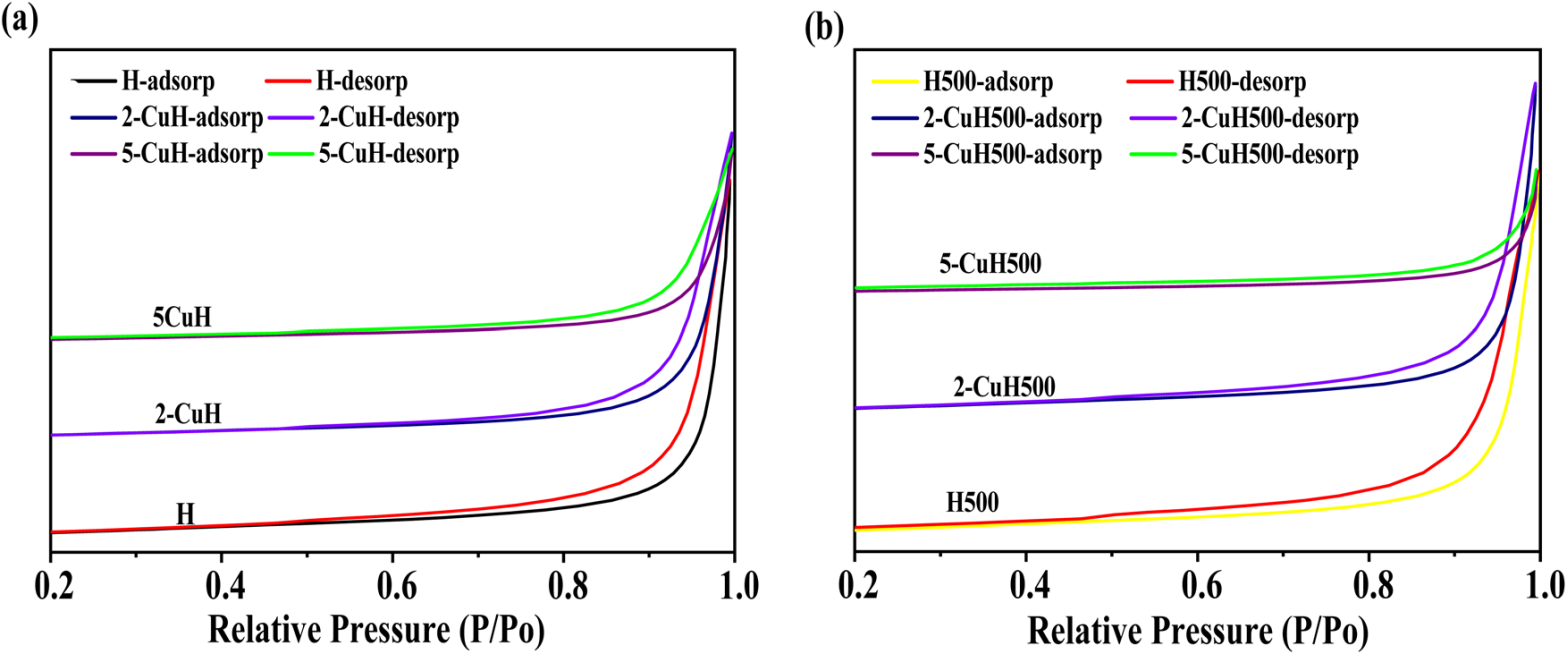
Nitrogen adsorption–desorption isotherms of the synthesized materials of noncalcined materials (a) and calcined materials (b).

The nitrogen adsorption/desorption isotherm analysis results for six representative material samples are presented in Table 4 and Fig. 5.

**Table 4:** BET surface area, pore aiameter, and pore volume of the synthesized materials

Sample	BET surface area (m^2^ g^−1^)	Pore diameter (nm)	Pore volume (cm^3^ g^−1^)
H	67.76	27.6	0.542
2-CuH	57.57	27.9	0.402
5-CuH	41.39	23.4	0.242
H500	58.88	26.4	0.389
2-CuH500	50.05	27.5	0.345
5-CuH500	26.34	16.7	0.109

**Fig. 6:**
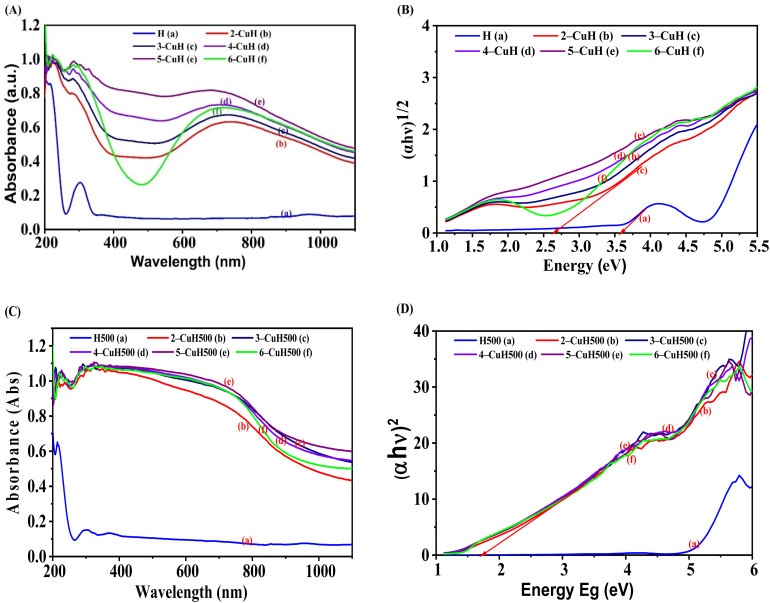
UV-Vis DRS spectra of the material samples: uncalcined samples (A), calcined samples (C), and Tauc plots used to determine the band gap energy of the material samples: uncalcined samples (B), calcined samples (D).

The nitrogen adsorption–desorption isotherms (BET) presented in Fig. 6 reveal that all six synthesized materials exhibit type IV isotherms with H3 hysteresis loops according to the IUPAC classification, which is characteristic of mesoporous materials.^9,31^ The narrow hysteresis loops observed in the isotherms indicate that the BET surface area and pore volume of the analyzed materials are relatively low. In particular, the 5-CuH500 sample shows a more extended nitrogen adsorption–desorption curve over the entire relative pressure range (*P*/*P*_0_ from 0 to 1.0), suggesting the coexistence of both mesoporous and microporous structures in this sample.

The data presented in Table 3 and Fig. S1 (SI) showed that both the BET surface area and pore volume of the non-calcined samples (H, 2-CuH, and 5-CuH) and the calcined samples (H500, 2-CuH500, and 5-CuH500) decreased significantly as the Cu/Al molar ratio increases from 0 to 5/3. Additionally, the average pore diameter also tended to decrease with increasing Cu content. This reduction in surface area and pore volume was attributed to the deterioration of the hydrotalcite-like layered structure due to Cu^2+^ incorporation, leading to partial or complete structural collapse, particularly after thermal treatment at high temperatures. These findings are consistent with the XRD and SEM analyses and agree with the results reported in previous studies.^22,26^

#### 3.1.6. UV-Vis diffuse reflectance spectra (DRS) of the studied materials

The shift in the light absorption edge and the band gap energy of the material samples was determined through their UV-Vis diffuse reflectance spectra (UV-Vis DRS). Fig. 6A and C present the UV-Vis DRS spectra of uncalcined and calcined material samples, respectively. In Fig. 6A, the uncalcined H and CuH samples exhibit a prominent absorption peak at approximately 220 nm and another absorption peak at around 320 nm, which are attributed to charge transfer transitions from O^2−^ to metal ions in tetrahedral coordination.^32^ Additionally, the CuH samples display a strong absorption peak at approximately 730 nm, which is ascribed to d–d charge transfer transitions of Cu^2+^ ions in an octahedral field. The absorption edge of the H sample lies in the ultraviolet region, consistent with previous reports.^28^ However, the Cu^2+^-modified H samples show a significant red shift in the absorption edge into the visible light region (*λ*_max > 400 nm) as the Cu/Al molar ratio increases from 0 to 5/3. Furthermore, the 6-CuH sample shows a blue shift in *λ*_max compared to 4-CuH and 5-CuH samples.^33,34^

Fig. 6C illustrates the UV-Vis DRS spectra of samples calcined at 500 °C. After calcination, the H500 sample also displays two major absorption peaks at around 220 and 320 nm, similar to the uncalcined H sample. However, the peak intensity at 320 nm in the H500 sample is significantly lower than that of the uncalcined H. In contrast, the calcined CuH samples exhibit a marked red shift of the absorption edge into the visible region. The UV-Vis DRS spectra of calcined samples differ markedly from those of uncalcined ones. This difference can be attributed to the formation of CuO nanoparticles in the materials after calcination, which strongly affects the absorption edge and significantly reduces the band gap energy.

Fig. 6B and D show the Tauc plots used to determine the band gap energy of the materials. The H and CuH samples follow an indirect allowed transition model (Tauc method with exponent *m* = 1/2), whereas the calcined H500 and CuH500 samples correspond to a direct allowed transition (*m* = 2).^35^ Based on these results, the band gap energy (*E*_g_) of the synthesized materials was calculated and summarized in Table S1 (see SI). A significant decrease in *E*_g_ was observed with increasing Cu^2+^ ion content, suggesting that Cu^2+^-modified materials are likely to exhibit enhanced photocatalytic activity under visible light irradiation.

## 4. The adsorption capacity of phenol red in the dark by the materials

**Fig. 7:**
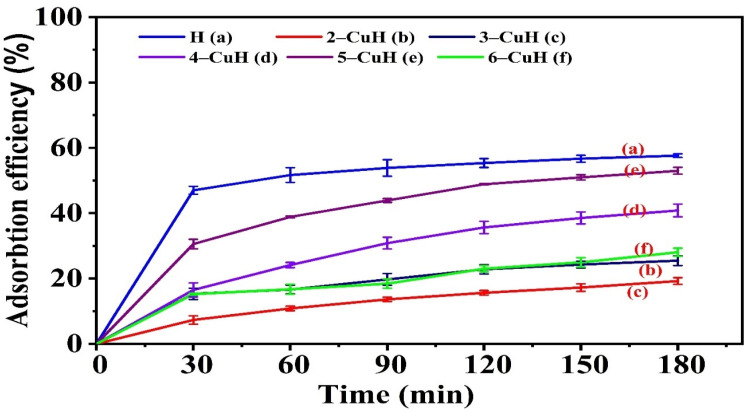
Adsorption efficiency of 100 ppm phenol red (PR) over time on non-calcined materials H and *n*-CuH (*n* = 2–6).

The research team investigated the adsorption capacity of phenol red (PR) using the procedure outlined in Section II (2.5.1). Table S2 (see SI) and Fig. 7 and 9 present the corresponding results.

### 4.1. Adsorption efficiency of non-calcined samples for phenol red in the dark

**Fig. 8:**
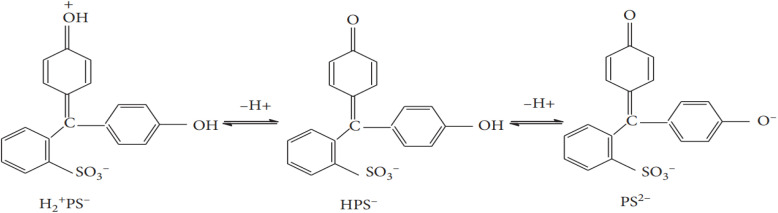
Speciation of phenol red in different environments.^36^

Several non-calcined and calcined samples effectively adsorbed 100 ppm PR from an aqueous solution, as shown in Fig. 7. Most materials reached adsorption equilibrium within 150 minutes. Among the non-calcined hydrotalcites, samples H, 4-CuH, and 5-CuH exhibited the highest adsorption capacities. The order of adsorption performance was: H > 5-CuH > 4-CuH > 6-CuH > 3-CuH > 2-CuH. The anionic nature of PR, which dissociates into forms such as H_2_^+^PS^−^, HPS^−^, and PS^2−^ in water (Fig. 8), largely explains this behaviour.

Electrostatic attraction between PR anions and metal cations on the brucite-like layers, as well as anion exchange with interlayer carbonate (CO_3_^2−^), likely facilitated the adsorption. A decrease in BET surface area may have further influenced the efficiency. The MgAlCO_3_ and CuMgAlCO_3_ materials share similar structures with ZnAlCO_3_ and CuZnAlCO_3_ and also adsorbed PR effectively. However, some MgAlCO_3_- and CuMgAlCO_3_-based samples achieved higher PR adsorption efficiency than those in the Zn-based series.^9^ Their greater BET surface areas likely contributed to this improved performance.

### 4.2. Adsorption efficiency of calcined samples for phenol red in the dark

**Fig. 9:**
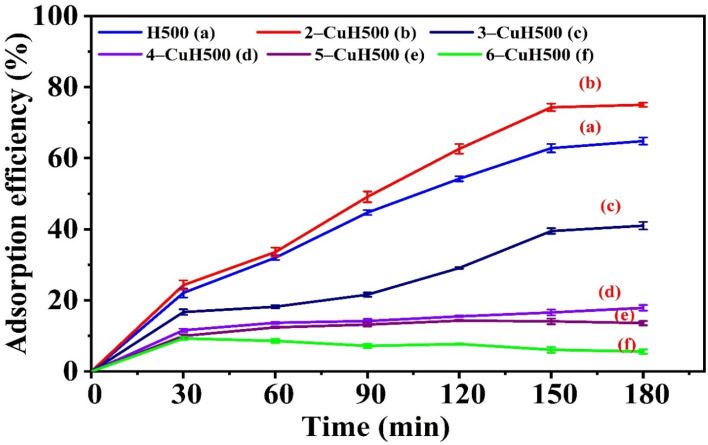
Adsorption efficiency of 100 ppm phenol red (PR) over time on calcined materials H500 and *n*-CuH500 (*n* = 2–6).

The hydrotalcite-based materials calcined at 500 °C also exhibited good adsorption capacity for 100 ppm phenol red (PR) in aqueous solution (Table S3 (see SI) and Fig. 9). However, the equilibrium adsorption time was 150 minutes for the 3 samples of H500, 2-CuH500, 3-CuH500, but only 60 minutes for the remaining 3 samples of 4-CuH500, 5-H500, 6-CuH500. The adsorption efficiency followed a decreasing order as: 2-CuH500 > H500 > 3-CuH500 > 4-CuH500 > 5-CuH500 > 6-CuH500. The H500 and 2-CuH500 samples, especially, showed better adsorption performance for 100 ppm PR compared to the non-calcined H and 5-CuH samples.

In addition to factors such as the layered hydrotalcite structure, BET surface area, pore volume, pore diameter, and electrostatic interactions between PR anions and metal cations, the so-called memory effect played a significant role in enhancing the adsorption performance of calcined samples such as H500, 2-CuH500, and 3-CuH500.^26,37^ Based on the evaluation of PR (100 ppm) adsorption under dark conditions, we further investigated the photocatalytic activity of the synthesized materials by assessing their ability to degrade PR. The study also identified optimal conditions for PR degradation, including illumination time, Cu^2+^ content in the material, PR concentration, and solution pH, and evaluated the materials' potential for application in the treatment of textile dyeing wastewater.

### 4.3. The interactions between Cu^2+^-modified Mg–Al materials and phenol red

The improved adsorption performance of Cu^2+^-modified Mg–Al LDHs results from multiple interacting mechanisms. Incorporating Cu^2+^ into the LDH structure increases surface acidity and introduces additional active binding sites, which enhances electrostatic attraction and complexation with phenol red (PR) molecules.^22^ Cu^2+^ ions also promote π–π interactions and may engage in redox processes, further supporting non-covalent bonding with the aromatic rings of PR.^38^

In addition, surface hydroxyl groups, interlayer anions, and the modified Cu^2+^ sites collectively contribute to the adsorption process through ion exchange, hydrogen bonding, and coordination interactions.^9^ These synergistic mechanisms significantly boost the adsorption capacity and efficiency of the Cu^2+^-modified materials compared to unmodified Mg–Al LDHs.

## 5. Photocatalytic degradation of phenol red under UV-Visible light

### 5.1. Effects of illumination time and Cu/Al molar ratio in the series of synthesized materials

**Fig. 10:**
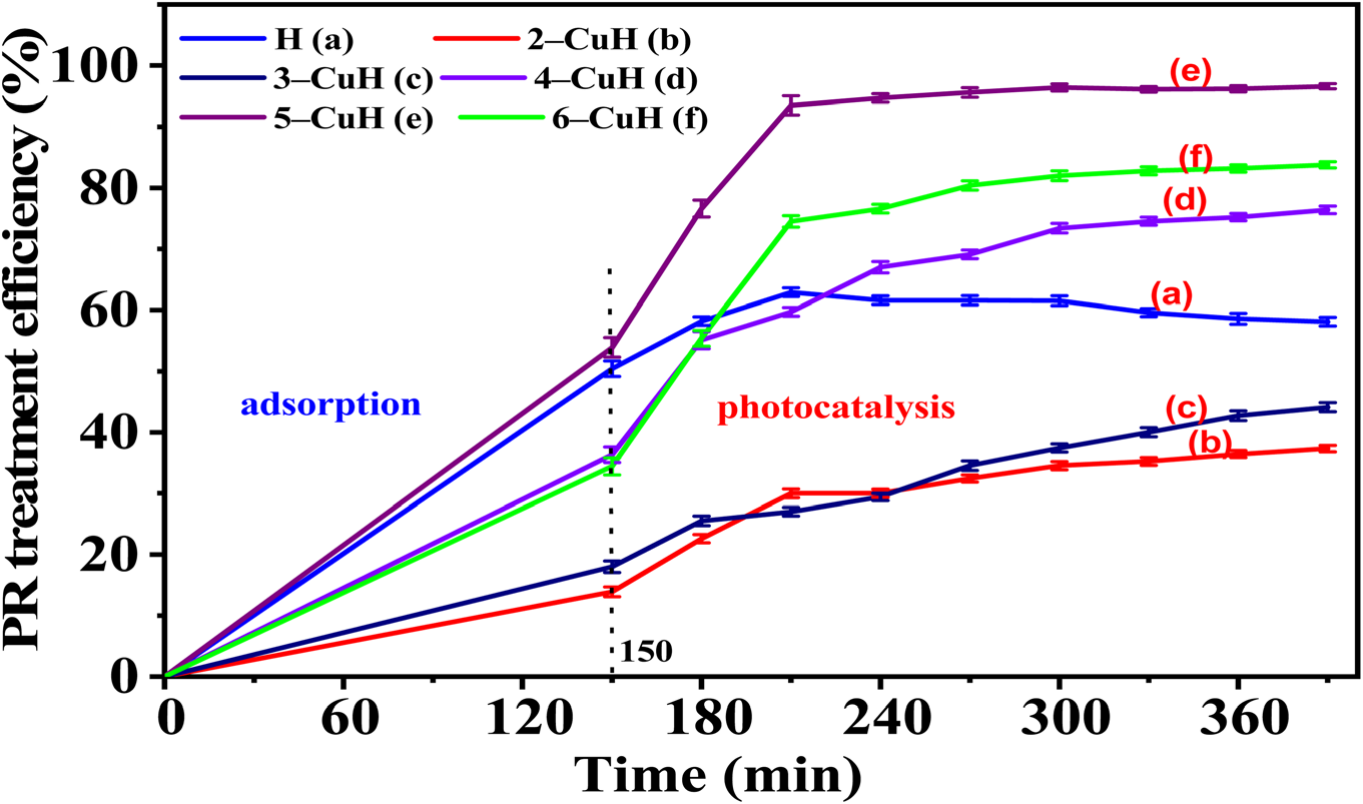
Treatment efficiency of 100 ppm phenol red (PR) over time on H and *n*-CuH samples (*n* = 2–6).

**Fig. 11:**
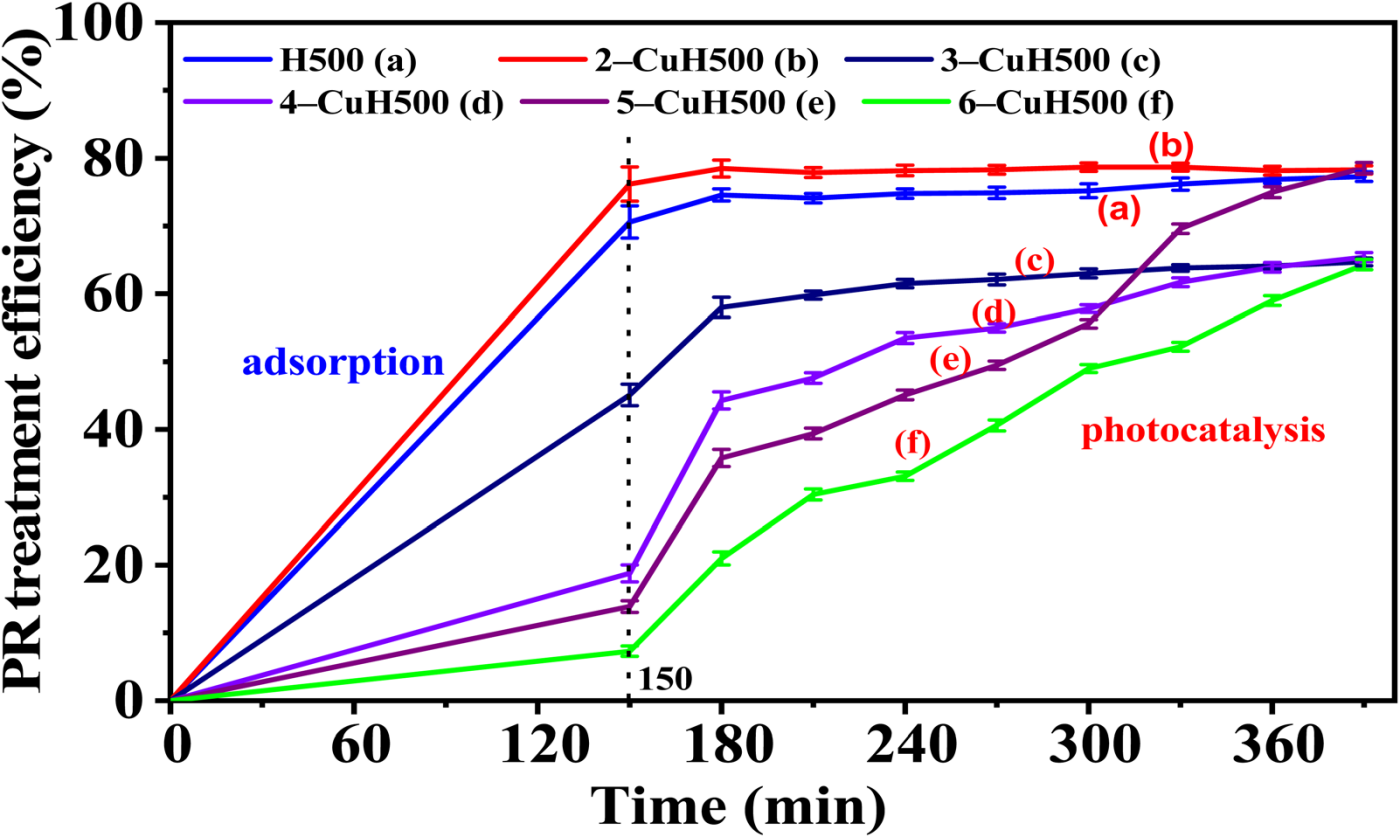
The treatment efficiency of 100 ppm phenol red (PR) over time on calcined samples H500 and *n*-CuH500 (*n* = 2–6).

The PR degradation capacity of the material samples was investigated following the procedures described in Sections 2.4.1 and 2.4.2. The results are presented in Tables S4, S5 (see SI) and Fig. 10 and 11.

#### 5.1.1. Non-calcined samples

As shown in Table S4 (SI) and Fig. 10, the degradation efficiency of 100 ppm phenol red (PR) on non-calcined samples increased with illumination time. After 150 minutes of dark stirring, adsorption efficiencies on H, 4-CuH, 5-CuH, and 6-CuH were 50.4%, 36.3%, 53.9%, and 34.4%, respectively. Upon illumination, PR degradation on the H sample increased slightly after 60 minutes, then gradually decreased over the next 180 minutes, indicating that adsorption is the dominant mechanism. This is attributed to the H sample's high BET surface area, pore diameter, and pore volume, which favor PR adsorption.

However, the H sample had a large bandgap energy (*E*_g_) and mainly absorbed UV light, resulting in negligible PR (100 ppm) degradation. In contrast, Cu^2+^-modified MgAlCO_3_ samples exhibited excellent photocatalytic performance. Among the non-calcined modified materials, 5-CuH showed the highest photocatalytic activity, achieving a total treatment efficiency of approximate 94% within 60 minutes of illumination, including about 40% from photocatalytic degradation. Based on Table S4 and Fig. 10, the photocatalytic activity decreased in the order: 5-CuH > 6-CuH > 4-CuH > 3-CuH > 2-CuH > H. The high activity under 30 W LED light is attributed to the synergy between appropriate bandgap energy, high BET surface area, Cu^2+^ active sites, illumination time, and the presence of H_2_O_2_ as an oxidizing agent. The PR degradation mechanism can be explained by the following reactions.^9,22,39^

7 Cu^2+^ − MgAl + *hυ* → Cu^2+^ − MgAl (e^−^, h^+^)

8 h^+^ + H_2_O_2_ → 2˙OH

9 H_2_O_2_ + e^−^ → ˙OH + OH^−^

10 OH˙ + PR → colorless reduction products

11 h^+^ + PR → colorless oxidation products

Compared to CuZnAlCO_3_ materials with Cu/Al molar ratios of 3/3 and 3.5/3 (samples CuH-3.0 and CuH-3.5), the 5-CuH sample exhibited significantly higher photocatalytic activity. This suggests that a Cu/Al molar ratio of 5/3 may be optimal for obtaining materials with superior photocatalytic performance.^9^

#### 5.1.2. Calcined samples

For the calcined material series (H500, *n*-CuH500), results from Table S5 (SI) and Fig. 11 indicate that the samples 4-CuH500, 5-CuH500, and 6-CuH500 exhibited good photocatalytic degradation of 100 ppm phenol red. In these samples, 5-CuH500 showed the highest photocatalytic activity, with a combined adsorption and degradation efficiency of 78.6% after 150 minutes of dark stirring and 240 minutes of illumination. In contrast, samples H500, 2-CuH500 primarily showed adsorption activity, with photocatalytic degradation efficiencies below 7%. The H500 sample had a high band gap energy (5.2 eV), which was not suitable for visible light excitation. Although the 2-CuH500 sample had a narrow band gap energy (1.73 eV), it was suitable for visible light excitation. However, due to the highest PR adsorption efficiency (76.2%), the PR molecules adsorbed on the material surface will reduce the absorption of excitation light, reducing the number of photogenerated e^−^ – h^+^ pairs, leading to low photocatalytic activity of this 2-CuH500 sample. In addition, the 3-CuH500 sample has shown the ability to photocatalytically decompose PR (photocatalytic decomposition efficiency of about 20% after 240 minutes of illumination).

Compared to the calcined samples, the uncalcined samples 4-CuH, 5-CuH, and 6-CuH demonstrated higher PR (100 ppm) treatment efficiency than their respective calcined counterparts (4-CuH500, 5-CuH500, and 6-CuH500) under identical conditions and with equal Cu^2+^ content. This result indicates that the layered hydrotalcite structure and the incorporation of Cu^2+^ ions into the brucite-like lattice play a crucial role in enhancing the photocatalytic activity of uncalcined materials. Based on these findings, the two representative samples, 5-CuH (uncalcined) and 5-CuH500 (calcined) were selected to further investigate the effects of PR concentration and pH on photocatalytic performance, as well as their potential for treating real textile dyeing wastewater (mat weaving effluent).

### 5.2. Effects of phenol red concentration on the catalytic activity of the synthesized materials

**Fig. 12:**
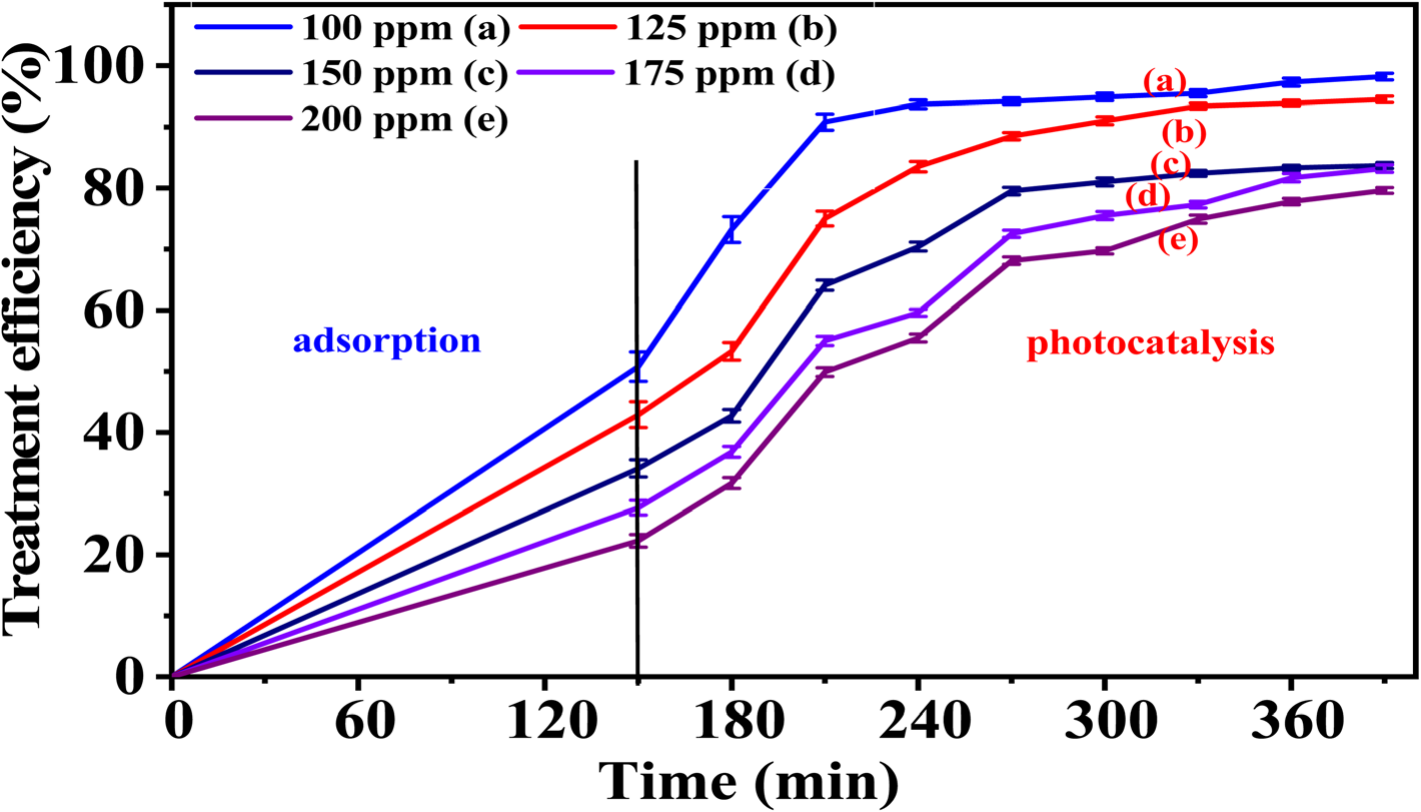
Graph illustrating the treatment efficiency of PR at different concentrations using the 5-CuH material.

**Fig. 13:**
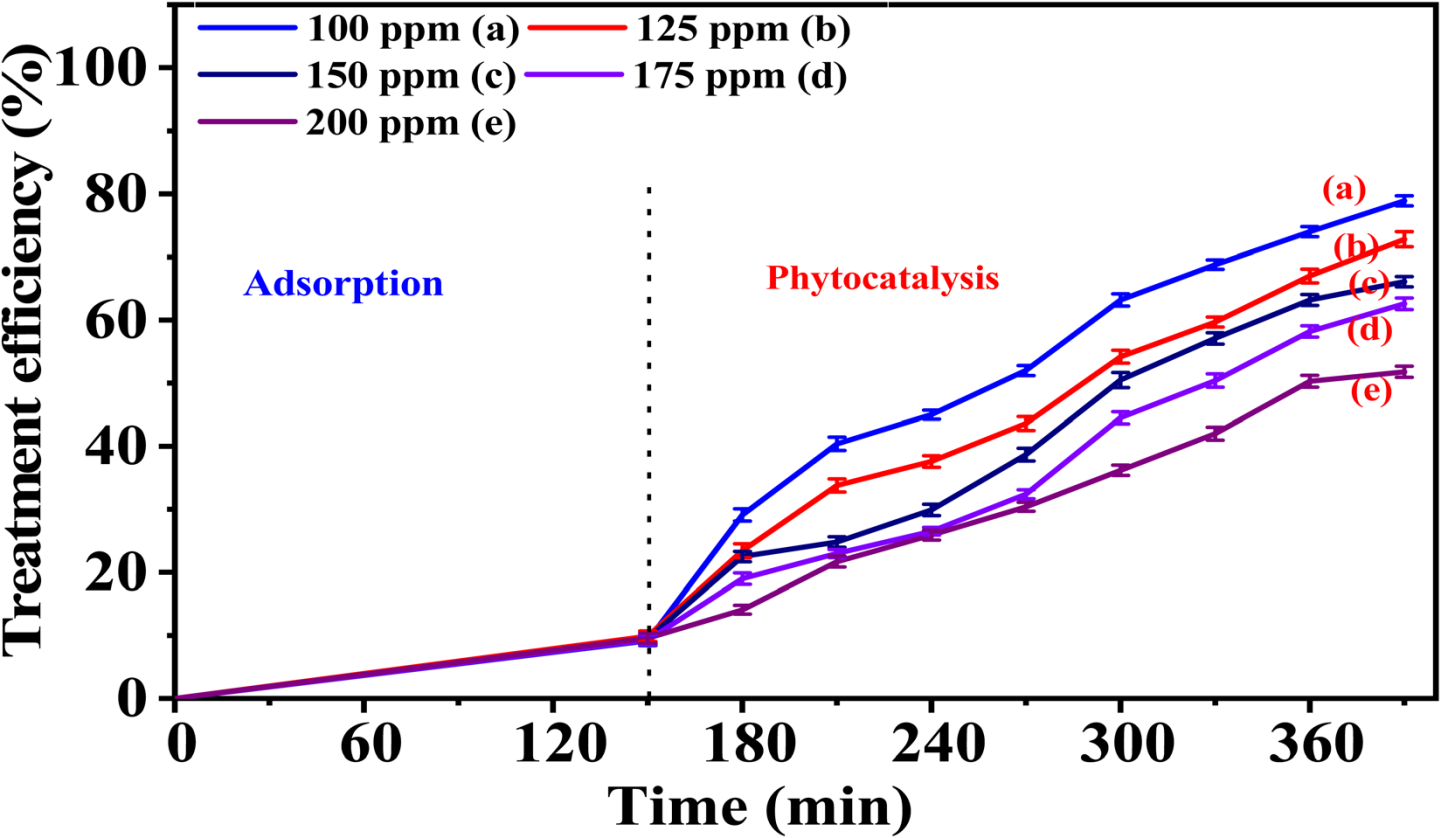
The treatment efficiency of phenol red (PR) at various concentrations using the 5-CuH500 material.

Two representative samples, 5-CuH and 5-CuH500, corresponding to the non-calcined and calcined material series, respectively, were selected to investigate the effect of PR concentration on their catalytic activity. High concentrations of PR (100, 125, 150, 175, and 200 ppm) were used in the experiments. The obtained results are presented in Tables S6, S7 (SI), Fig. 12, and 13.

The results presented in Table S6 and Fig. 12 showed that the treatment efficiency of PR gradually decreased as the initial PR concentration increased from 100 to 200 ppm. At a concentration of 100 ppm, the treatment efficiency exceeded 90% after just 60 minutes of illumination. However, for higher concentrations (150, 175, and 200 ppm), the illumination time required to reach approximately 83% treatment efficiency extended to 240 minutes. Despite the reduced efficiency at higher concentrations, the 5-CuH material still demonstrated a strong photocatalytic capacity, capable of degrading PR at concentrations up to 200 ppm. This performance surpasses that of previously reported CuH-3.0 and CuH-3.5 materials,^9^ highlighting the superior photocatalytic activity of 5-CuH and its potential for treating wastewater containing high concentrations of phenol red.

For the calcined sample 5-CuH500, the results presented in Table S7 and Fig. 13 revealed a similar degradation trend to that of the uncalcined 5-CuH sample and aligned with the general rule that increasing the pollutant concentration leads to a decrease in reaction rate and overall degradation efficiency.

When comparing PR degradation efficiencies at corresponding concentrations between the two samples containing the same amount of Cu^2+^ ions (5-CuH and 5-CuH500), the photocatalytic activity of 5-CuH was significantly higher than that of 5-CuH500 under identical experimental conditions. This further confirms the crucial role of the layered structure, the coordination of Cu^2+^ ions with O^2−^, and the isomorphic substitution of Cu^2+^ for Mg^2+^ in the brucite-like lattice in enhancing the photocatalytic performance of the material.

**Fig. 14:**
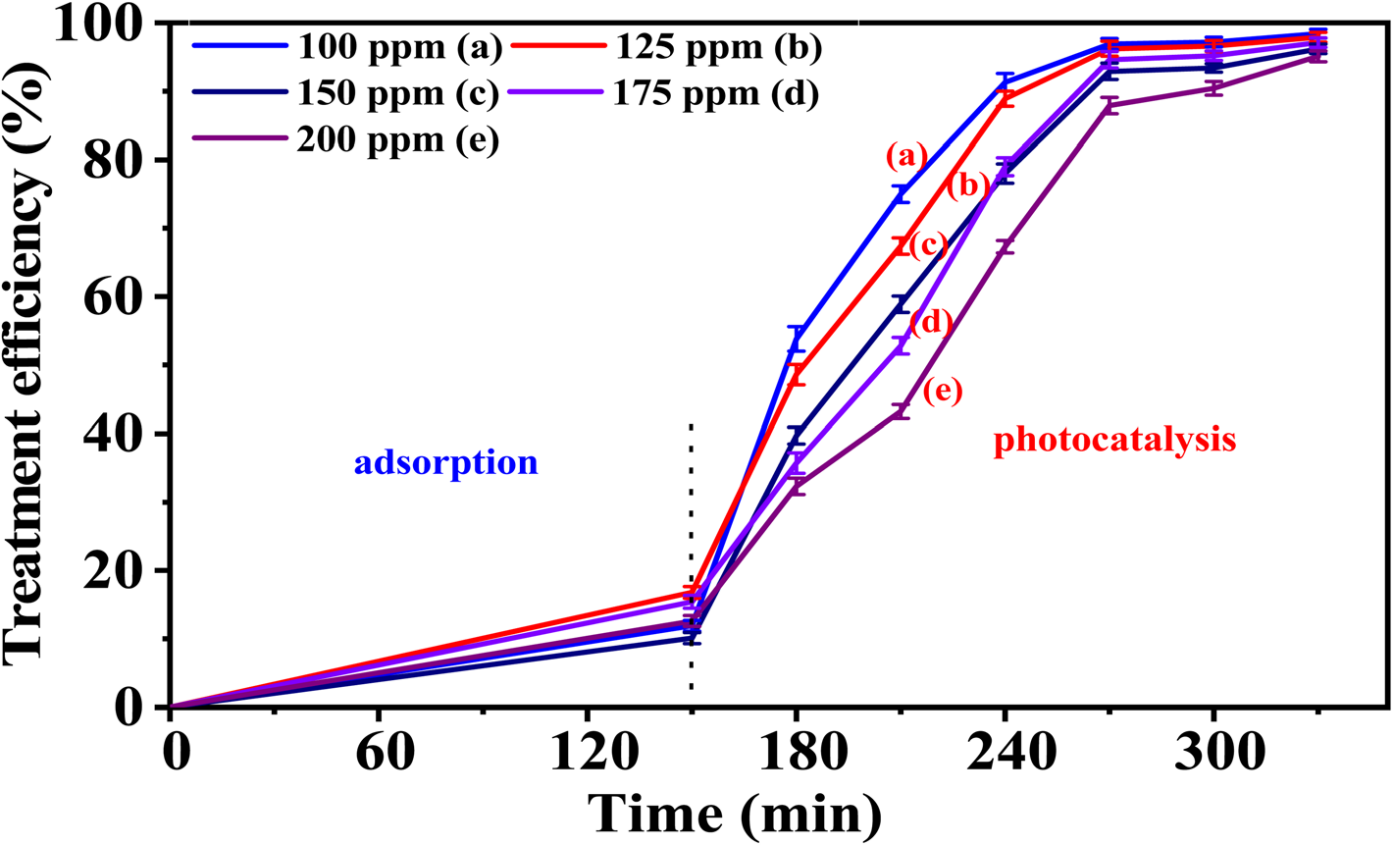
The treatment efficiency of phenol red at various concentrations by 5-CuH500 at pH 3.0.

After investigating the influence of pH on the catalytic activity of the material (Section 2.4.4), we examined the treatment efficiency of phenol red at various initial concentrations using the 5-CuH500 sample at pH 3.0. The results are presented in Table S8 (SI) and Fig. 14.

Results presented in Table S8 and Fig. 14 reveal that the 5-CuH500 sample exhibited significantly enhanced photocatalytic activity under acidic conditions (pH = 3.0). When comparing the degradation of PR at 100 ppm under two different pH conditions (initial pH = 4.15 and pH = 3.0), the sample showed markedly improved performance at lower pH. At pH 3.0, the material achieved over 91.3% treatment efficiency for 100 ppm PR within just 90 minutes of LED light exposure. The 5-CuH500 material also demonstrated strong photocatalytic performance at high pollutant concentrations. It degraded 200 ppm of phenol red with an efficiency of 82.5% after 180 minutes of illumination.

These findings confirm that both 5-CuH and 5-CuH500 can effectively degrade high concentrations of phenol red (up to 200 ppm) under mild experimental conditions (30 W LED light, room temperature and pressure). The excellent catalytic activity of both materials highlights their strong potential for practical wastewater treatment applications.

### 5.3. Effects of solution pH on the catalytic activity of synthesized materials

Two materials, 5-CuH and 5-CuH500, were selected to investigate the effect of solution pH on the treatment of phenol red (PR) at concentrations of 150 ppm and 100 ppm, respectively.

#### 5.3.1 5-CuH material

**Fig. 15:**
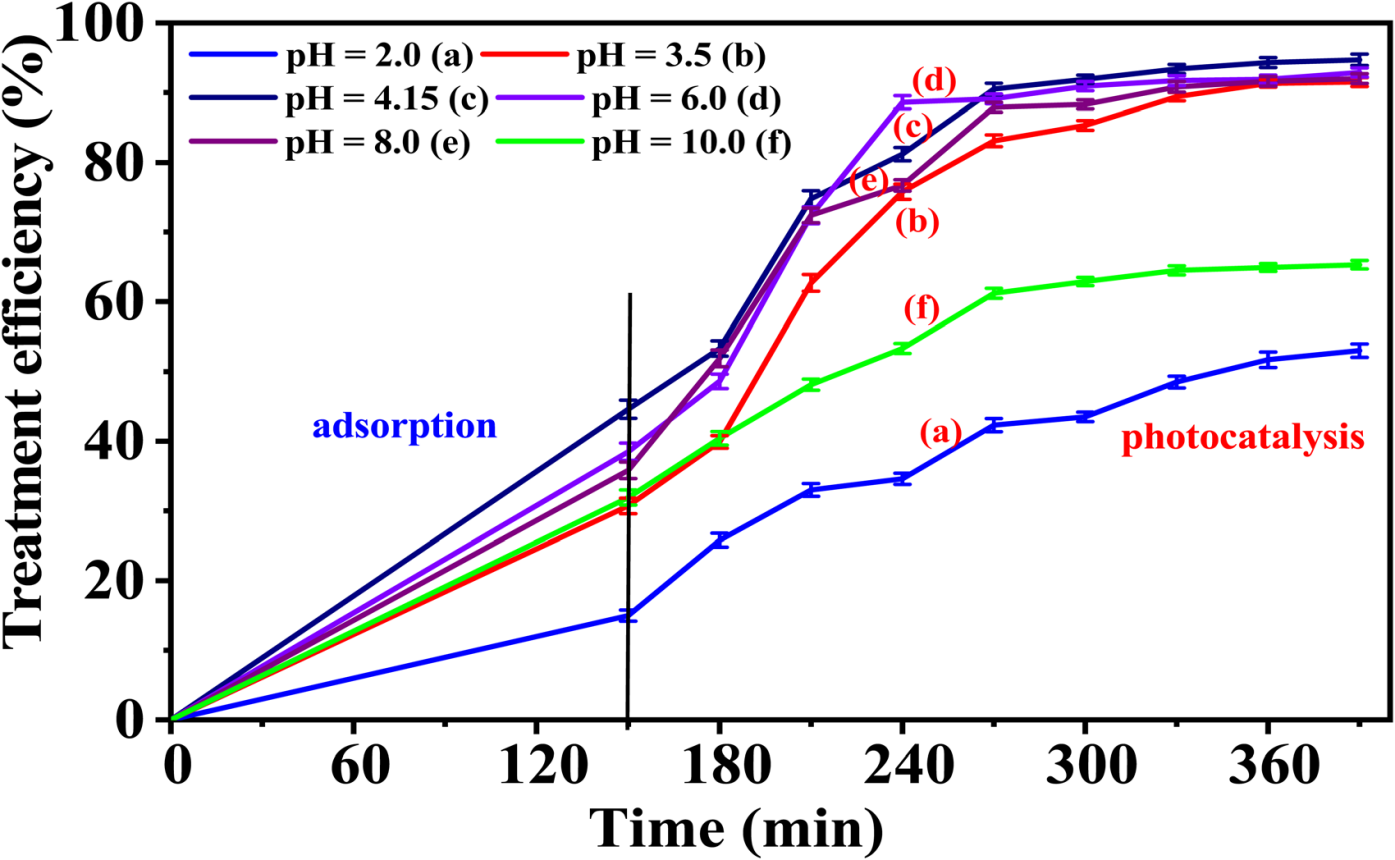
The treatment efficiency of 150 ppm phenol red over time by 5-CuH at different pH levels.

The effect of solution pH on the photodegradation of phenol red (PR) at concentrations of 150 ppm of the 5-CuH material was shown in Table S9 and Fig. 15. The photocatalytic activity of 5-CuH remained high across a broad pH range of 3.5 to 8.0. However, the highest treatment efficiency for 150 ppm PR occurred within the slightly acidic range of pH 4.15 to 6.0, indicating that weakly acidic conditions are optimal for PR treatment using 5-CuH.

Lowering the pH below 4.15 resulted in a gradual decline in degradation efficiency. Although 5-CuH maintained high activity at pH 3.5, a strong acidic environment (pH 2.0) significantly reduced its efficiency due to structural degradation of the material under highly acidic conditions (pH < 3.0).^9^

As the pH increased from 4.15 to 6.0, 8.0, and 10.0, the catalytic activity initially improved but then declined. This decrease at higher pH values is likely due to competition between PR anions and hydroxide ions (OH^−^) for adsorption sites, along with increased solution viscosity, which hinders catalytic performance. These findings align well with previous studies.^9^

#### 5.3.2 5-CuH500 material

**Fig. 16:**
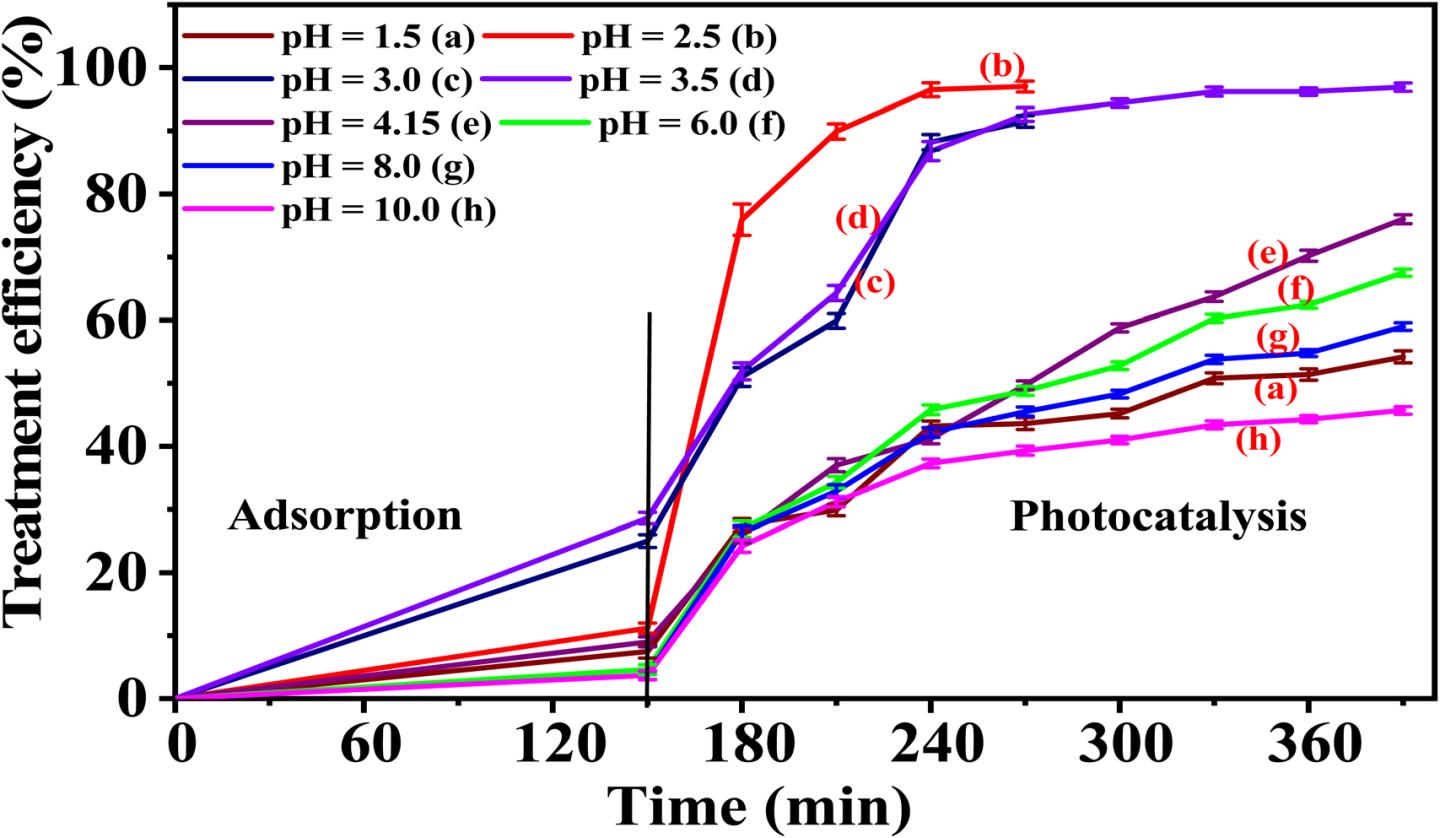
The treatment efficiency of 100 ppm phenol red over time using 5-CuH500 at different pH values.

Table S10 (SI) and Fig. 16 indicate the effects of pH values on the degradation of PR at various times under the presence of 5-CuH500. Fig. 16 shows that environmental pH significantly influenced the photocatalytic activity of the 5-CuH500 sample. Under strongly acidic conditions (pH 1.5–2.5), the material's activity sharply declined compared to its performance at the initial pH of PR 100 ppm solution (pH = 4.15). This reduction is attributed to the gradual structural degradation of the material during light exposure, which occurs more rapidly at lower pH levels and leads to decreased catalytic efficiency.

In the pH range of 4.15 to 10.0, photocatalytic activity also showed a downward trend with increasing pH. However, reducing the pH from 4.15 to 3.5, 3.0, and 2.5 significantly enhanced the material's catalytic performance. Within the optimal range of pH 2.5–3.5, the treatment efficiency of 100 ppm PR reached approximately 97% within just 90 minutes of LED light irradiation at pH 2.5.

This enhanced activity may result from the partial dissolution of CuO, MgO, and Al_2_O_3_ under weakly acidic conditions, releasing metal ions such as Cu^2+^ that re-adsorb onto the material surface. These ions form Cu^2+^-CuMgAlCO_3_ catalytic centers, which boost the material's ability to convert H_2_O_2_ under LED light into more hydroxyl radicals (HO˙), thereby accelerating PR degradation.

### 5.4. Proposed mechanism for the photocatalytic degradation of phenol red by the synthesized materials

To elucidate the active species and photogenerated charge carriers involved in the photocatalytic degradation of phenol red (PR), various scavengers were employed to assess their influence on the degradation efficiency. Experiments were conducted using a PR concentration of 100 ppm, with the solution pH adjusted to 4.15 for the 6-CuH sample and 3.0 for the 5-CuH500 sample. Four parallel experimental conditions were applied: (1) without any scavenger, (2) with 2 mL of 1 mM EDTA-2Na (a hole (h^+^) scavenger), (3) with 2 mL of 10 mM isopropyl alcohol (a hydroxyl radical (˙OH) scavenger), and (4) with 2 mL of 1 mM *p*-benzoquinone (a superoxide radical (O_2_˙^−^) scavenger). Scavengers were introduced after the addition of 1.2 mL of 30% H_2_O_2_ and following a dark equilibration period to establish adsorption–desorption equilibrium.

**Fig. 17:**
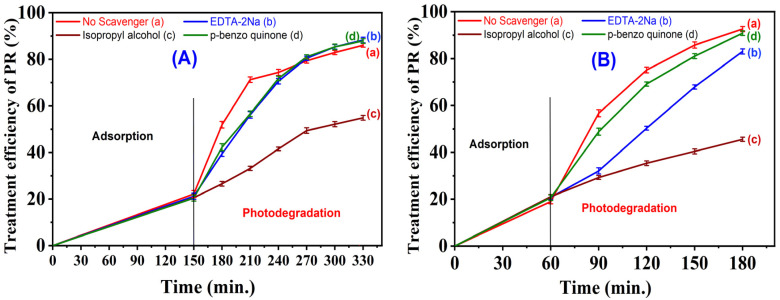
Degradation efficiency of 100 ppm phenol red (PR) using 6-CuH (A), and 5-CuH500 in the presence and absence of active species and hole scavengers (B).

For the 6-CuH material, the experimental results clearly indicated that hydroxyl radicals (˙OH) played the dominant role in the photocatalytic degradation of PR (Fig. 17A). In contrast, for the 5-CuH500 sample, both hydroxyl radicals (˙OH) and photogenerated holes (h^+^) were found to contribute significantly to the degradation process (Fig. 17B). These findings are consistent with previously reported studies^40,41^

The UV-Vis spectra of PR presented in Fig. S2C and D (SI) illustrate that the intensity of the characteristic absorption peak at 435 nm decreased more slowly over time when isopropyl alcohol was added to the reaction system. Under these conditions, the degradation efficiencies of 100 ppm PR after 180 and 120 minutes of irradiation were reduced to 54.8% and 45.5% for 6-CuH and 5-CuH500, respectively (Fig. 17A and B). These results confirm that isopropyl alcohol effectively inhibits the activity of hydroxyl radicals (˙OH), thereby slowing down the photocatalytic degradation of PR for both materials.

### 5.5. Role of H_2_O_2_ in the degradation of phenol red by the synthesized materials

To clarify the role of 30% H_2_O_2_ in generating hydroxyl radicals (˙OH) involved in the degradation of phenol red (PR), both 6-CuH and 5-CuH500 materials were investigated under five experimental conditions: (a) without catalyst, with H_2_O_2_ and under light irradiation, (b) with catalyst and light irradiation, without H_2_O_2_, (c) with catalyst, H_2_O_2_, and light irradiation, (d) with catalyst, without light (dark conditions), (e) with catalyst and H_2_O_2_, without light (dark conditions). The initial pH was maintained at 4.15 for 6-CuH and 3.0 for 5-CuH500 during these experiments.

**Fig. 18:**
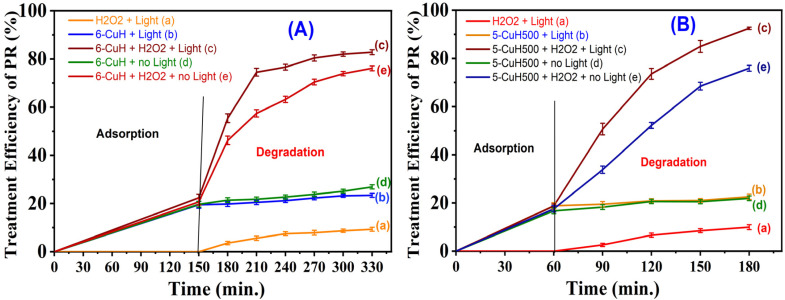
Degradation efficiency of 100 ppm phenol red (PR) using 6-CuH (A) and 5-CuH500 (B), respectively, under five different conditions.

The results indicated that PR degradation efficiency remained low in cases (a), (b), and (d) (Fig. 18A and B). Specifically, in the condition where only H_2_O_2_ and light were applied, the degradation efficiency of 100 ppm PR reached only 9.3% at pH 4.15 and approximately 10% at pH 3.0. This suggests that the 30 W LED light source alone could activate H_2_O_2_ to a limited extent, producing only a small quantity of ˙OH radicals and resulting in poor PR degradation. Similarly low efficiencies were observed when only the catalyst was used under light or in the dark.

In contrast, when both the catalyst and H_2_O_2_ were present, the PR degradation efficiency significantly increased over time. Notably, the combination of catalyst, H_2_O_2_, and LED light irradiation produced substantially higher degradation efficiencies compared to the same system under dark conditions. This confirms that the synergistic interaction of all three components, photocatalyst, H_2_O_2_, and light, leads to a more rapid and abundant generation of hydroxyl radicals. Moreover, in the absence of light, the combined effect of the catalyst and H_2_O_2_ suggests a heterogenous Fenton-like reaction mechanism contributing to ˙OH formation.

These findings demonstrate that the simultaneous presence of catalyst, H_2_O_2_, and light markedly enhances the degradation of 100 ppm PR. Under optimized conditions, the degradation efficiency reached approximately 83% after 330 minutes using 6-CuH at pH 4.15, and about 93% after 180 minutes using 5-CuH500 at pH 3.0.

The UV-Vis absorption spectra of PR solutions (Fig. S3C, D and E (SI)) showed a characteristic peak at 435 nm, with only a gradual decrease in intensity over time when using H_2_O_2_ alone without catalyst (Fig. S3C), or in the presence of only the catalyst under light (Fig. S3D and E (SI)). These results emphasize the crucial role of H_2_O_2_ in facilitating PR degradation when used in combination with the synthesized materials.

## 6. Investigation of the treatment efficiency for sedge mat dyeing textile wastewater

### 6.1. Evaluation of dye degradation capability of the synthesized materials

**Fig. 19:**
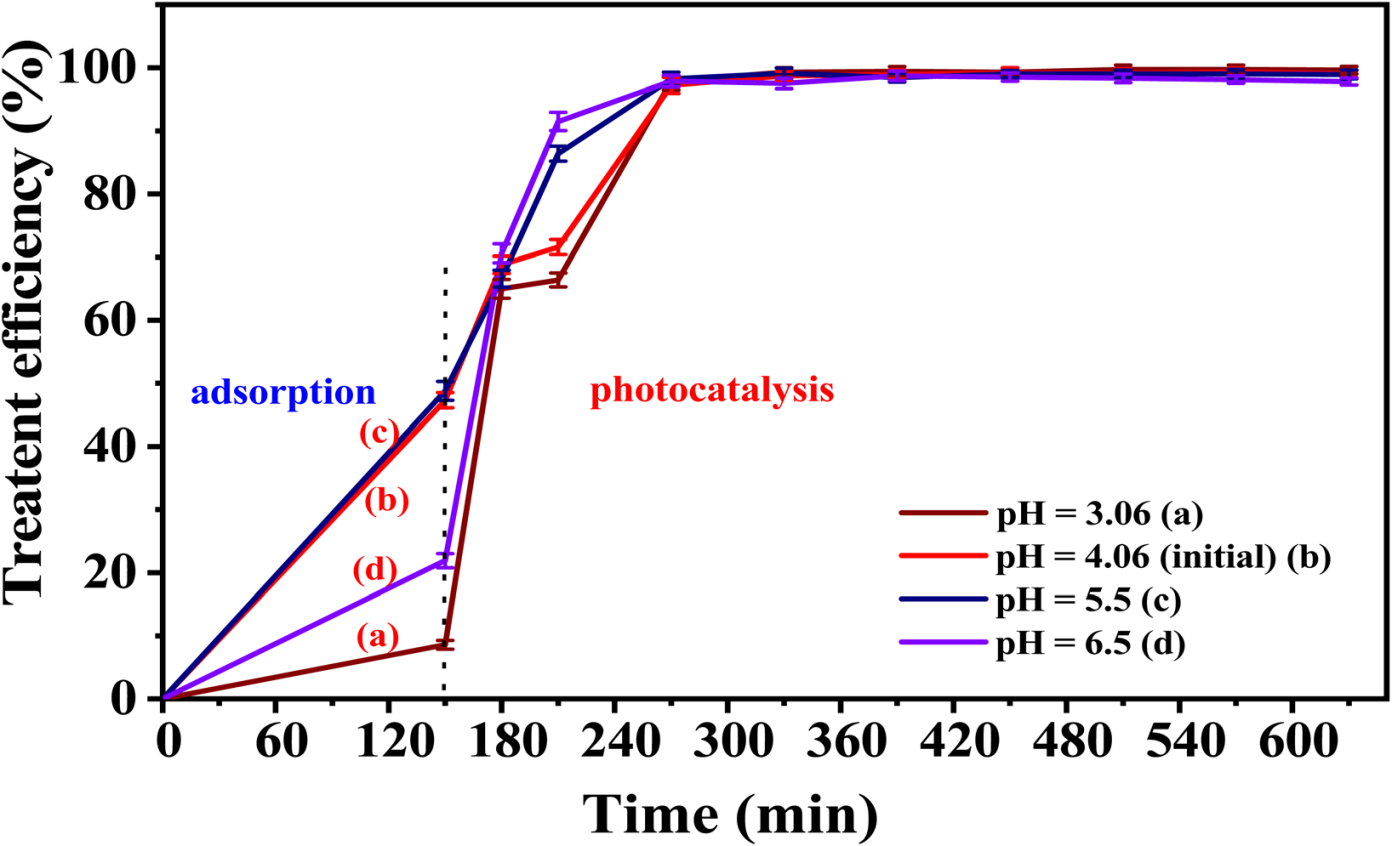
Time-dependent treatment efficiency of dye compounds in diluted mat-weaving textile wastewater treated with the 5-CuH material in the presence of 30% H_2_O_2_ under visible light irradiation.

**Fig. 20:**
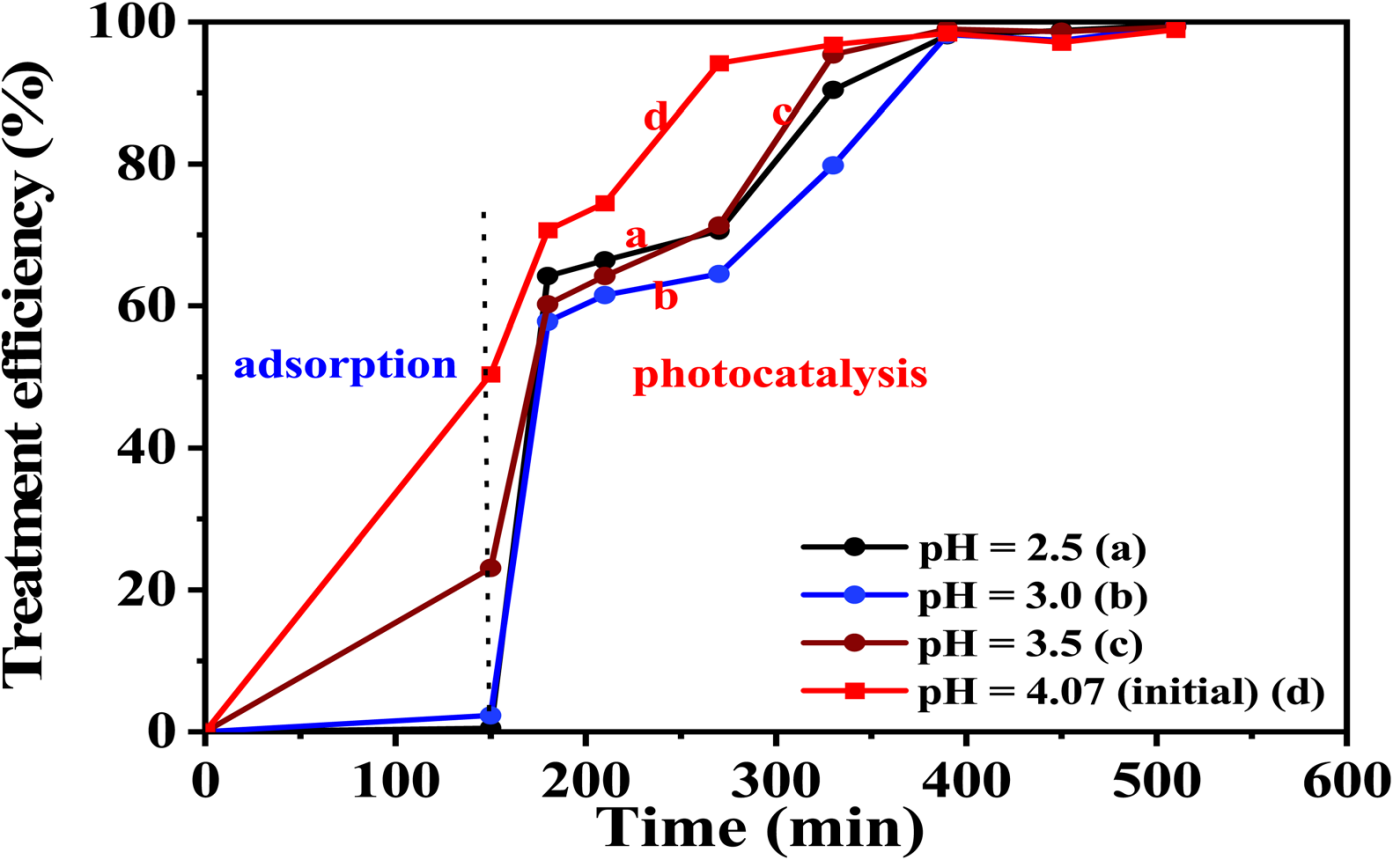
Time-dependent treatment efficiency of dye compounds in diluted mat-weaving textile wastewater treated with the 5-CuH500 material in the presence of 30% H_2_O_2_ under visible light irradiation.

This study investigates the treatment efficiency of synthetic materials for textile wastewater generated from the mat weaving industry. The wastewater, characterized by a dark red color, was sourced from a household production site in Dong Bang village, An Le commune, Quynh Phu district, Thai Binh province of Vietnam. After a tenfold dilution with distilled water, the samples 5-CuH and 5-CuH500, combined with 5 mL of 30% H_2_O_2_, were applied to assess the degradation of dyes and persistent organic compounds. The treatment results are presented in Tables 5 and 6, and illustrated in Fig. 19 and 20.

**Table 5:** The treatment efficiency (%) of dye compounds present in diluted textile wastewater from the mat weaving industry, treated using the 5-CuH catalyst and 30% H_2_O_2_ under visible light irradiation^a^

Time (min)	The treatment efficiency of dyes in wastewater for sample 5-CuH (%) at different pH levels							
	pH = 3.06		pH = 4.06 (initial)		pH = 5.5		pH = 6.5	
	Abs	H%	Abs	H%	Abs	H%	Abs	H%
0	1.715	0	1.715	0	1.715	0	1.715	0
150 ad	1.568	8.6	0.904	47.3	0.878	48.8	1.339	21.9
180	0.6	65.0	0.535	68.8	0.572	66.6	0.504	70.6
210	0.577	66.4	0.487	71.6	0.234	86.4	0.145	91.5
270	0.036	97.9	0.0474	97.2	0.0301	98.2	0.0361	97.9
330	0.0126	99.3	0.0226	98.7	0.0159	99.1	0.0431	97.5
390	0.0107	99.4	0.0191	98.9	0.0281	98.4	0.023	98.7
450	0.0113	99.3	0.0139	99.2	0.0174	99.0	0.0264	98.5
510	0.0047	99.7	0.0162	99.1	0.0173	99.0	0.0289	98.3
570	0.0056	99.7	0.0126	99.3	0.018	99.0	0.033	98.1
630	0.0074	99.6	0.0166	99.0	0.0195	98.9	0.0383	97.8

a ad: adsorption; pH (initial): pH value of the original (untreated) dye wastewater.

**Table 6:** The treatment efficiency of colorants present in rush mat dyeing wastewater by the 5-CuH500 material^a^

Time (min)	The treatment efficiency of dyes in wastewater by the 5-CuH500 material (%)							
	pH = 2.5		pH = 3.0		pH = 3.5		pH = 4.07 (initial)	
	Abs	H%	Abs	H%	Abs	H%	Abs	H%
0	1.715	0.0	1.715	0.0	1.715	0.0	1.715	0.0
150 ad	1.707	0.5	1.676	2.3	1.318	23.1	0.851	50.4
180	0.614	64.2	0.724	57.8	0.682	60.2	0.503	70.7
210	0.577	66.4	0.66	61.5	0.614	64.2	0.438	74.5
270	0.504	70.6	0.608	64.5	0.492	71.3	0.099	94.2
330	0.164	90.4	0.346	79.8	0.079	95.4	0.055	96.8
390	0.0321	98.1	0.0301	98.2	0.0166	99.0	0.0279	98.4
450	0.0207	98.8	0.0453	97.4	0.0232	98.6	0.0492	97.1
510	0.0076	99.6	0.0111	99.4	0.0126	99.3	0.0197	98.9

a ad: adsorption; pH (initial): pH value of the original (untreated) dye wastewater.

Based on the obtained results, both 5-CuH and 5-CuH500 materials exhibited strong capabilities in degrading colorants present in rush mat dyeing wastewater. The 5-CuH sample achieved over 97% treatment efficiency within just 90 minutes of light irradiation across the investigated pH ranges. In comparison, the 5-CuH500 sample also reached degradation efficiencies exceeding 90%, albeit within a slightly longer irradiation period of 120 to 180 minutes.

These findings clearly confirm that both materials possess effective photocatalytic activity toward the treatment of organic colorants in dyeing wastewater. Their high performance highlights the practical applicability of these synthesized materials in the removal of industrial textile effluents.

The UV-Vis spectra of colorants in the dyeing wastewater varied over time during the adsorption and photocatalytic degradation processes, as shown in Fig. S4. At pH 6.5, the 5-CuH sample exhibited low adsorption efficiency, reaching only about 22% after 150 minutes. In contrast, the 5-CuH500 material achieved approximately 50% under the same conditions. Consequently, the intensity of the maximum absorption peak at 552 nm after 150 minutes was significantly lower for 5-CuH500 than for 5-CuH. A broad and intense absorption peak appeared at around 447 nm at the initial stage of the wastewater. However, this peak was rapidly degraded within 30 minutes of light irradiation on both materials. The absorption peak at 552 nm also declined sharply after approximately 120 minutes. Visually, the color of the solution significantly faded following centrifugation to remove solids. To accurately evaluate the mineralization of organic compounds, the samples were further irradiated for up to 10 hours. COD measurements were subsequently performed to assess the mineralization efficiency of the 5-CuH and 5-CuH500 materials.

### 6.2. Evaluation of the mineralization efficiency of organic compounds in mat-dyeing wastewater by synthesized materials

**Fig. 21:**
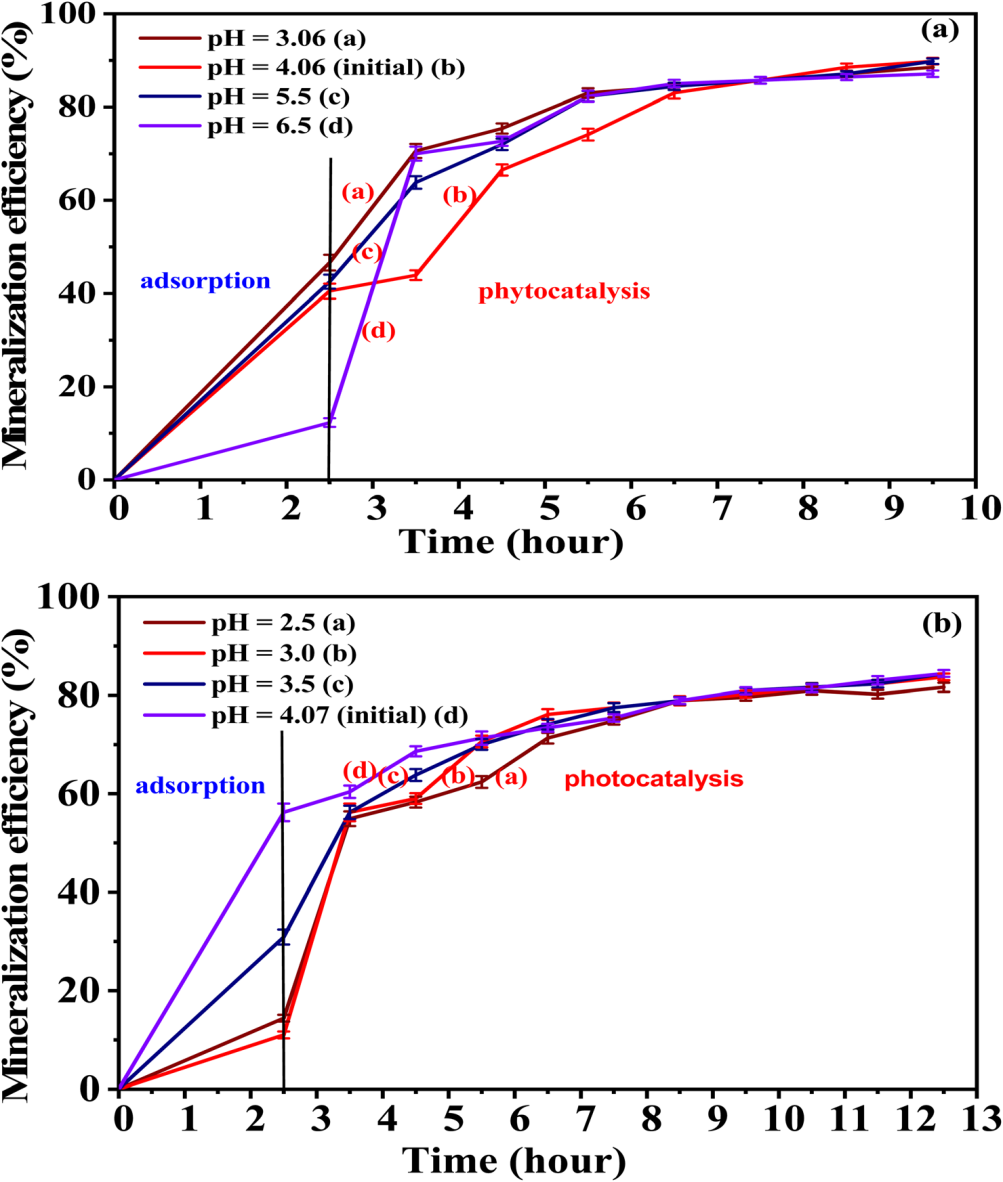
Mineralization efficiency of organic compounds in mat-dyeing wastewater treated with 5-CuH (a) and 5-CuH500 (b).

We simultaneously conducted experiments to determine dye degradation efficiency and measure the COD of wastewater after different irradiation periods. The results are presented in Tables S11, S12, and Fig. 21(a and b).

The obtained results show that a strong correlation was observed between the decrease in dye absorbance and the reduction in COD values. As dye absorbance intensity declined, COD levels also decreased. However, the COD reduction occurred at a significantly slower rate than the decrease in absorbance. After approximately 120 minutes of irradiation, dye degradation efficiencies exceeded 90% for both 5-CuH and 5-CuH500 materials. In contrast, the mineralization efficiency of organic compounds reached only 67–75% for 5-CuH and 58–69% for 5-CuH500.

These findings indicate that 5-CuH500 exhibited lower mineralization performance at the respective pH levels compared to 5-CuH. Moreover, after 10 hours of removal, 5-CuH reduced the COD of the dyeing wastewater from 486 to 49.3 mg L^−1^, meeting column A of the industrial wastewater discharge standard. In comparison, 5-CuH500 showed a slower mineralization rate, reducing COD from 486 to 76 mg L^−1^ after 12.5 hours.

Overall, both 5-CuH and 5-CuH500 demonstrated high efficiency in degrading dye compounds and effectively mineralizing refractory organics in mat-dyeing wastewater. These results suggest that the tested materials offer promising potential for advanced textile wastewater treatment applications.

## 7. Comparative analysis

Organic pollutant elimination like phenol red (PR) continues to be a priority area in advanced material development. Amongst the prospective materials, Cu^2+^-modified Mg–Al layered double hydroxides (LDHs) have been found quite promising, with improved performance in most significant parameters.

Compared with state-of-the-art persulfate-activated catalysts such as CoFe_2_O_4_, although the latter may be pushed to rapid degradation rates (greater than 95% in 20 minutes), it does so at the cost of greater operational cost and complexity from the need to utilize external oxidants.^42^ Cu–Mg–Al LDHs, on the other hand, offer a greener, self-activating alternative that integrates both adsorption and *in situ* catalytic degradation, demonstrating their effectiveness and affordability for practical application.^43^

Conclusively, Cu^2+^-modified Mg–Al LDHs outperform conventional and state-of-the-art adsorbents in pollutant treatment by a large margin. Their superior adsorption capacity, kinetics, stability across a broad pH range, and decent reusability render them highly prospective for wastewater treatment applications at the practical level. Additionally, their dual-mode treatment mechanism not only enhances their efficiency but also preserves cost-effectiveness and environmental sustainability relative to more complex systems.^44,45^

## 8. Stability and reusability of the materials

To evaluate the stability and reusability of the photocatalytic materials, two highly active samples, namely 6-CuH and 5-CuH500, were selected for investigation. Given that the pH values of the phenol red (PR) solutions used (100 ppm) were 4.15 and 3.0, optimal for the performance of 6-CuH and 5-CuH500, respectively, the experiments were conducted under these conditions.

**Fig. 22:**
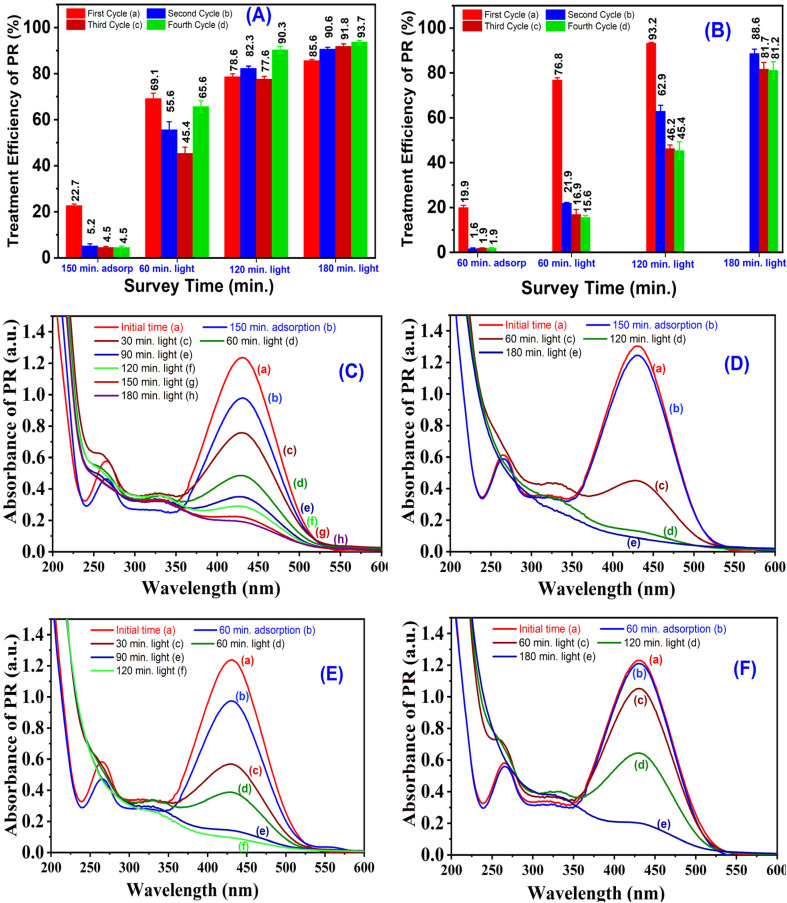
Degradation efficiency of 100 ppm phenol red (PR) over time at pH 4.15 using the 6-CuH material (A), and at pH 3.0 using the 5-CuH500 material (B); UV-Vis absorption spectra of PR (100 ppm) after treatment with 6-CuH in the first (C) and fourth (D) reuse cycles; and with 5-CuH500 in the first (E) and fourth (F) reuse cycles.

The treatment efficiencies of 100 ppm PR solution after four successive reuse cycles of each material are presented in Fig. 22A and B. For the 6-CuH sample, a noticeable decrease in adsorption efficiency was observed after the first use, dropping sharply from 22.7% to 5.2% after 150 minutes of dark stirring. In subsequent cycles, the adsorption efficiency remained relatively stable, with only a slight decrease from 5.2% to 4.5%. Interestingly, the treatment efficiency of PR showed a modest increase over time, rising from 85.6% in the first cycle to 93.7% in the fourth cycle after 180 minutes of light irradiation.

In contrast, the 5-CuH500 material exhibited a marked decline in both adsorption capacity and photocatalytic activity across four reuse cycles. Initially, the material achieved an adsorption efficiency of 19.9% after 60 minutes of dark stirring, which significantly decreased to 1.6%, 1.9%, and 1.9% in the second, third, and fourth cycles, respectively. Under optimal conditions at pH 3.0, 5-CuH500 demonstrated a high treatment efficiency of 93.2% within just 120 minutes of illumination during the first use. However, this efficiency dropped substantially during the subsequent cycles at both 60 and 120 minutes of irradiation. When the irradiation time was extended to 180 minutes, the treatment efficiencies slightly recovered to 88.6%, 81.7%, and 81.2% for the second, third, and fourth uses, respectively. These findings are supported by UV-Vis absorption spectra of PR solutions shown in Fig. 22C and 22F

The observed reduction in adsorption and photocatalytic performance for both materials can be attributed to the gradual leaching of Cu^2+^ ions into the solution over time. The dissolution of copper ions likely results in a reduced number of catalytic active sites, consequently diminishing the generation of hydroxyl radicals (˙OH), which are essential for effective photocatalytic degradation. Furthermore, Cu^2+^ leaching may also alter the structural integrity of the materials, potentially affecting their long-term photocatalytic behavior.

**Fig. 23:**
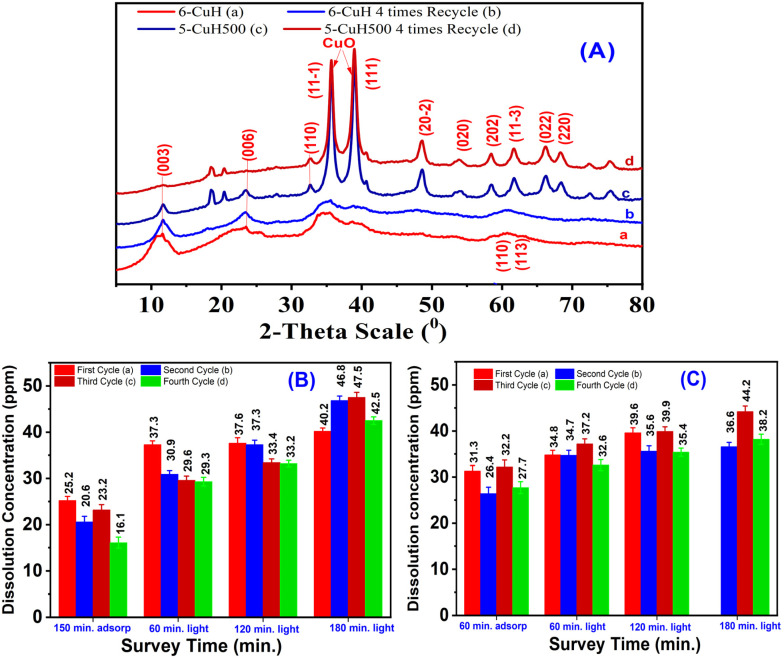
(A) XRD patterns of 6-CuH and 5-CuH500 materials before and after four reuse cycles; (B) concentration of dissolved Cu^2+^ ions in the solution from the 6-CuH sample after four reuse cycles; (C) concentration of dissolved Cu^2+^ ions in the solution from the 5-CuH500 sample after four reuse cycles.

The XRD patterns of the 6-CuH and 5-CuH500 samples after four consecutive uses, compared with their respective pristine forms, are presented in Fig. 23A. As shown, no significant changes were observed in the characteristic hydrotalcite-like layered structure or the oxide phases of either material after repeated use. These results indicate that both 6-CuH and 5-CuH500 retain their layered double hydroxide-like structural features and oxide phase compositions even after four reuse cycles.

Moreover, the concentration of Cu^2+^ ions leached into the solution increased progressively with each reuse cycle. In general, the total Cu^2+^ concentration detected after 180 minutes of irradiation was consistently higher for the 6-CuH sample (Fig. 23B) compared to the 5-CuH500 sample (Fig. 23C). This suggests that the Cu(OH)_2_ phase present in 6-CuH is more prone to dissolution than the CuO phase in 5-CuH500, despite the higher solution pH used for 6-CuH (pH = 4.15) compared to 5-CuH500 (pH = 3.0). Nevertheless, the photocatalytic performance of both materials remained relatively stable after three reuse cycles, achieving treatment efficiencies of 93.7% and 81.2% for 6-CuH and 5-CuH500, respectively, after 180 minutes of light exposure.

## 9. Practical application of materials for textile dyeing wastewater treatment

### 9.1. Potentials for practical application in wastewater treatment

Cu^2+^-doped Mg–Al layered double hydroxides (LDHs) have been discovered to hold tremendous potential for the treatment of real-scale wastewater, such as the treatment of organic pollutants like phenol red (PR). The authors have synthesized these materials using co-precipitation, which allows for scale-up and large-scale production. They have higher adsorption capacity and enhanced photocatalytic activity, allowing for effective treatment of dye-contaminated wastewater. For example, Cu^2+^-doped Zn–Al LDH was reported to achieve more than 90% degradation of PR under LED irradiation and H_2_O_2_, indicating excellent efficiency. This is an indication that Cu^2+^-modified Mg–Al LDHs would complement current treatment systems to handle diversified contaminants.

### 9.2. Environmental benefits and limitations of the materials

Since Cu^2+^-modified Mg–Al LDHs work effectively to treat wastewater, there is a requirement to critically examine their environmental benefits and potential limitations.

#### 9.2.1. Advantages

##### 9.2.1.1. High efficiency

The materials are effective in removing heavy metals and organic matters from wastewater due to their high photocatalytic and adsorption activities.

##### 9.2.1.2. Reusability

They possess structural integrity and efficiency issues with various challenges of reuse cycles, minimizing wastage and the replacement requirement.

#### 9.2.2. Limitation

##### 9.2.2.1. Stability problems

Long-term performance with varying environmental conditions remains uncertain and requires investigation.

##### 9.2.2.2. Metal leaching

Cu^2+^ ions can leach into treated water, producing leach hazards of contamination. Effective stabilization and post-treatment are needed by researchers to overcome this.

##### 9.2.2.3. Cost factors

Despite easy synthesis, material cost and raw material cost and mass production cost must be assessed for economic feasibility.

In general, Cu^2+^-modified Mg–Al LDHs possess considerable environmental benefits, including high treatment efficiency, and reusability. Nevertheless, material stability, prevention of metal leaching, and cost assessment remain essential to practical application.

## 10. Conclusions

(i) Cu^2+^-modified Mg–Al hydrotalcites showed strong potential for adsorbing and photocatalytically degrading phenol red (PR). Structural analyses (XRD, EDX, FT-IR, SEM) confirmed Cu^2+^ incorporation *via* isomorphic substitution, resulting in reduced crystallinity and mesoporous structures. Uncalcined samples (*e.g.*, 5-CuH) maintained layered morphology, enabling about 94% PR treatment through electrostatic attraction, π–π interactions, and ion exchange. Visible-light photocatalysis was enhanced by Cu^2+^-induced bandgap narrowing (*E*_g_ ≈ 2.1–3.1 eV), with 5-CuH achieving approximately 40% degradation in 60 minutes. Calcined samples (*e.g.*, 5-CuH500) formed CuO nanoparticles but had reduced efficiency due to structural collapse, though they performed better in acidic conditions. Both materials reduced COD by over 90% in real textile wastewater, validating their dual-function mechanism.

(ii) Recommendations for future study future studies should optimize the Cu/Al ratio and investigate co-doping to prevent pore collapse. Enhancing structural stability during calcination and under extreme pH is critical to preserving material integrity. Minimizing Cu leaching through surface passivation is essential to reduce secondary pollution. Finally, assessing scalability, cost-efficiency, and reusability under real wastewater conditions will support practical application.

(iii) Environmental and industrial relevance these materials provide an efficient, sustainable solution for textile wastewater treatment under mild conditions (30 W LED, ambient temperature). They combine high adsorption with visible-light photocatalysis, reducing dependence on UV systems. To enable large-scale use, Cu leaching control and scalable synthesis remain key challenges. Overall, Cu–Mg–Al hydrotalcites present a viable alternative to conventional adsorbents and catalysts in eco-friendly wastewater treatment.

## Author contributions

Conceptualization: V.·N.·V.; methodology: V.·N.·V. and T. H. T. P.; software: T. T. H.·P. and T. H. L.; validation: V.·N. V. and T. T. H.·P.; data curation: V.·N.·V., T. T. H.·P. and T. H. L.; writing original draft preparation, T. X. V. and V.·N.·V.; writing – review and editing, V. T. X. visualization: V.·N.·V. and T. X. V. All authors have read and agreed to the published version of the manuscript.

## Conflicts of interest

The authors declare no conflicts of interest.

## Supplementary Material

RA-015-D5RA02645H-s001

## Data Availability

All data obtained have been included in the manuscript and/or SI and are available from the corresponding author upon reasonable request. Additional data related to this paper may be requested from the corresponding author at xuanvt@tnus.edu.vn. Additional figures and tables supporting the adsorption experiments and material characterization results. See DOI: https://doi.org/10.1039/d5ra02645h.
