# Supplementary material for: Enhanced removal of phenol red from water by Cu(ii)-modified Mg–Al composites via adsorption and photocatalysis
Source: RSC Adv. 2025 Aug 11;15(35):28500–23. doi: 10.1039/d5ra02645h (PMC12376985; doi:10.1039/d5ra02645h)
Supplement: RA-015-D5RA02645H-s001 [file RA-015-D5RA02645H-s001.pdf]

Supplementary Information (SI) for RSC Advances.
This journal is © The Royal Society of Chemistry 2025

SUPPLEMENTARY INFORMATION

Title: Enhanced Removal of Phenol Red from Water by Cu(II)-Modified Mg-Al Composites via Adsorption and Photocatalysis

(Table S1-S11)

Table S1. Band gap energy (Eg) of the material samples determined by the Tauc method

Nr	Sample	Band gap energy (E_g) (eV)
1	H	3.58
2	2-CuH	2.63
3	3-CuH	2.55
4	4-CuH	2.09
5	5-CuH	1.95
6	6-CuH	2.63
7	H500	5.2
8	2-CuH500	1.73
9	3-CuH500	1.70
10	4-CuH500	1.65
11	5-CuH500	1.58
12	6-CuH500	1.70

Table S2. Adsorption efficiency of 100 ppm Phenol Red (PR) in the dark by the series of H and CuH materials (non-calcined samples).

Time (min)	Adsorption efficiency for PR 100 ppm on the Non-calcined samples (%)					
	**H**	**2–CuH**	**3–CuH**	**4–CuH**	**5–CuH**	**6–CuH**
0	0	0	0	0	0	0
30	47.0	7.3	15.3	16.5	30.6	15.2
60	51.7	10.8	16.6	24.1	38.8	16.7
90	53.8	13.6	19.7	30.8	43.9	18.4
120	55.3	15.6	22.8	35.6	48.9	23.0
150	56.7	17.2	24.3	38.5	50.9	24.9

180	57.6	19.2	25.5	40.8	53.0	28.0

Table S3. Adsorption efficiency of 100 ppm Phenol Red (PR) in the dark by the series of H500 and n-CuH500 materials (calcined samples).

Time (min)	Average adsorption efficiency for PR (100 ppm) on calcined samples (%)					
	**H500**	**2–CuH500**	**3–CuH500**	**4–CuH500**	**5–CuH500**	**6–CuH500**
0	0	0	0	0	0	0
30	22.1	24.3	16.7	11.6	10	9.3
60	32.0	33.6	18.2	13.7	12.4	8.6
90	44.7	49.1	21.6	14.2	13.2	7.2
120	54.2	62.6	29.1	15.5	14.3	7.7
150	62.8	74.3	39.5	16.6	14.1	6.1
180	64.5	75.1	41.2	17.2	13.6	5.6

Table S4. Treatment efficiency of 100 ppm Phenol Red (PR) on non-calcined H and n-CuH samples.

Time (min)	Mean treatment efficiency for PR 100 ppm on the non-calcined samples (%)					
	**H**	**2–CuH**	**3–CuH**	**4–CuH**	**5–CuH**	**6–CuH**
0	0	0	0	0	0	0
150_{ad}	50.4	13.9	18.0	36.3	53.9	34.4
180	58.2	22.6	25.5	55.1	76.6	55.4
210	62.9	30.0	27.0	59.7	93.5	74.5
240	61.6	30.0	29.4	67.0	94.7	76.6
270	61.6	32.4	34.5	69.1	95.6	80.4
300	61.5	34.5	37.4	73.4	96.4	82.0
330	59.6	35.2	40.0	74.53	96.1	82.8
360	58.6	36.4	42.7	75.2	96.2	83.2
390	58.1	37.3	44.1	76.4	96.6	83.8

Note 150_{ad}: 150 minutes adsorption

Table S5. The treatment efficiency of 100 ppm Phenol Red (PR) by calcined samples H500 and n-CuH500 (n = 2-6)

Time (min)	Mean treatment efficiency for PR (100 ppm) on calcined samples (%)					
	**H500**	**2–CuH500**	**3–CuH500**	**4–CuH500**	**5–CuH500**	**6–CuH500**
0	0	0	0	0	0	0
150ad	70.6	76.2	45.1	18.8	13.9	7.3
180	74.6	78.5	58.0	44.3	35.8	21.0
210	74.1	77.9	59.8	47.6	39.4	30.4
240	74.8	78.2	61.5	53.5	45.1	33.1
270	74.9	78.3	62.1	55.0	49.5	40.6
300	75.2	78.7	63.0	57.8	55.6	49.0
330	76.2	78.7	63.8	61.7	69.6	52.2
360	76.9	78.2	64.1	63.9	75.0	59.0
390	77.3	78.3	64.7	65.4	78.6	64.3

ad: adsorption

Table S6. Treatment efficiency of PR at different concentrations using the 5-CuH material

Time (min)	Mean treatment efficiency of PR at different concentrations using the 5-CuH material (%)				
	**100 ppm**	**125 ppm**	**150 ppm**	**175 ppm**	**200 ppm**
0	0	0	0	0	0
150ad	50.8	42.9	34.1	27.7	22.3
180	73.2	53.3	42.7	36.8	31.7
210	90.8	75.0	64.1	55.0	49.9
240	93.7	83.5	70.4	59.6	55.5
270	94.2	88.5	79.5	72.5	68.1
300	94.9	91.0	81.0	75.5	69.7
330	95.5	93.4	82.4	77.3	74.9
360	97.3	93.9	83.3	81.7	77.8
390	98.2	94.5	83.7	83.2	79.6

Table S7. Treatment efficiency of PR at different concentrations using the 5-CuH500 material

Time (min)	Mean treatment efficiency of PR at different concentrations using the 5-CuH500 (%)				
	**100 ppm**	**125 ppm**	**150 ppm**	**175 ppm**	**200 ppm**
0	0	0	0	0	0
150ad	9.2	9.9	9.5	9.3	9.6
30	29.1	23.4	22.5	19.0	14.1
60	40.4	33.8	24.8	23.0	21.7
90	45.0	37.6	29.9	26.5	25.9
120	52.0	43.6	38.7	32.4	30.4
150	63.2	54.2	50.5	44.5	36.2
180	68.8	59.7	57.1	50.4	42.0
210	74.0	67.0	63.2	58.2	50.3
240	78.9	72.8	66.1	62.6	51.8

ad: adsorption

Table S8. Treatment efficiency of Phenol Red at Different Concentrations by 5-CuH500 at pH 3.0

Time (min)	Mean treatment efficiency of Phenol Red at different concentrations by 5-CuH500 at pH 3.0 (%)				
	100 ppm	125 ppm	150 ppm	175 ppm	200 ppm
0	0	0	0	0	0
150ad	11.9	16.8	10.1	15.4	12.6
180	53.8	48.6	39.7	35.7	32.3
210	75.0	67.4	58.9	52.8	43.2
240	91.3	88.9	78	79	67.3
270	96.9	96.2	92.9	94.6	87.9
300	97.2	96.6	93.4	95.2	90.4
330	98.4	97.9	96.2	97.1	95.1

ad: adsorption

Table S9. Treatment efficiency of 150 ppm Phenol Red by 5-CuH at various pH levels

**Time (min)**	**Mean treatment efficiency of phenol red (150 ppm) at different pH levels (%)**					
	**pH = 2.0**	**pH = 3.5**	**pH = 4.15**	**pH = 6.0**	**pH = 8.0**	**pH = 10.0**
0	0	0	0	0	0	0
150ad	15.0	30.7	44.6	38.5	35.8	31.9
180	25.8	39.9	53.3	48.6	51.9	40.4
210	33.0	62.7	74.8	72.4	72.4	48.1
240	34.6	65.8	81.2	88.6	76.7	53.3
270	42.3	83.1	90.5	89.1	87.9	61.2
300	43.5	85.3	91.9	90.9	88.3	62.9
330	48.5	89.4	93.4	91.7	90.8	64.5
360	51.7	91.3	94.3	91.9	91.6	64.9
390	53.0	91.5	94.7	91.9	92.0	65.3

Table S10. Treatment efficiency of 100 ppm Phenol Red Using 5-CuH500 at Different pH Values

**Time (min)**	**Mean treatment efficiency of 100 ppm Phenol Red using 5-CuH500 at different pH values (%)**							
	**pH = 1.5**	**pH = 2.5**	**pH = 3.0**	**pH = 3.5**	**pH = 4.15**	**pH = 6.0**	**pH = 8.0**	**pH = 10.0**
0	0	0	0	0	0	0	0	0
150ad	7.5	11.2	25.0	28.6	9.0	4.6	3.7	3.7
180	27.5	75.9	51.0	51.9	25.9	26.9	26.3	24.2
210	29.9	89.9	59.9	64.3	37.0	34.3	32.9	31.2
240	43.2	96.5	88.2	86.8	41.2	45.8	42.2	37.3
270	43.6	97.0	91.5	92.6	49.7	48.8	45.5	39.3
300	45.2			94.4	58.8	52.8	48.3	41.0
330	50.8			96.2	63.7	60.3	53.8	43.4
360	51.4			96.2	70.2	62.4	54.8	44.3
390	54.2			96.9	76.0	67.5	59.0	45.7

ad: adsorption

Table S11. COD Values and Mineralization Efficiency of Refractory Organic Compounds in Mat-Dyeing Wastewater Treated with 5-CuH

**Time (hour)**	**Mean Mineralization Efficiency on 5-CuH (%)**							
	**pH = 3.06**		**pH = 4.06 (pH_{in})**		**pH = 5.5**		**pH = 6.5**	
	**COD**	**H%**	**COD**	**H%**	**COD**	**H%**	**COD**	**H%**
0	486.0	0.0	486.0	0.0	486.0	0.0	486.0	0.0
2.5 (ad)	259.3	46.6	289.3	40.5	279.3	42.5	426.0	12.3
3.5	142.7	70.6	272.7	43.9	176.0	63.8	146.0	70.0
4.5	119.3	75.4	162.7	66.5	136.0	72.0	132.7	72.7
5.5	82.7	83.0	126.0	74.1	86.0	82.3	86.0	82.3
6.5	76.0	84.4	82.7	83.0	76.0	84.4	72.7	85.0
7.5	69.3	85.7	69.3	85.7	69.3	85.7	69.3	85.7
8.5	62.7	87.1	56.0	88.5	62.7	87.1	66.0	86.4
9.5	56.0	88.5	49.3	89.8	49.3	89.8	62.7	87.1

ad: adsorption; pH_{in}: initial pH of the original solution

Table S12. COD Values and Mineralization Efficiency of Refractory Organic Compounds in Mat-Dyeing Wastewater Treated with 5-CuH500

Time (hour)	Mean Mineralization Efficiency of Organic Compounds on 5-CuH500 (%)							
	pH = 2.5		pH = 3.0		pH = 3.5		pH = 4.07 (pH_{in})	
	COD	H%	COD	H%	COD	H%	COD	H%
**0**	486.0	0	486.0	0	486.0	0	486.0	0
**2.5 (ad)**	416.0	14.4	432.7	11.0	336.0	30.9	212.7	56.2
**3.5**	219.3	54.9	212.7	56.2	212.7	56.2	192.7	60.4
**4.5**	202.7	58.3	199.3	59.0	176.0	63.8	152.7	68.6
**5.5**	182.7	62.4	142.7	70.6	146.0	70.0	139.3	71.3
**6.5**	139.3	71.3	116.0	76.1	126.0	74.1	129.3	73.4
**7.5**	122.7	74.8	109.3	77.5	109.3	77.5	119.3	75.4
**8.5**	102.7	78.9	102.7	78.9	102.7	78.9	102.7	78.9
**9.5**	99.3	79.6	96.0	80.2	92.7	80.9	92.7	80.9
**10.5**	92.7	80.9	89.3	81.6	89.3	81.6	90.3	81.4
**11.5**	96.0	80.2	86.0	82.3	86.0	82.3	82.7	83.0
**12.5**	89.3	81.6	79.3	83.7	76.0	84.4	76.0	84.4

ad: adsorption; pH_{in}: initial pH of the original solution

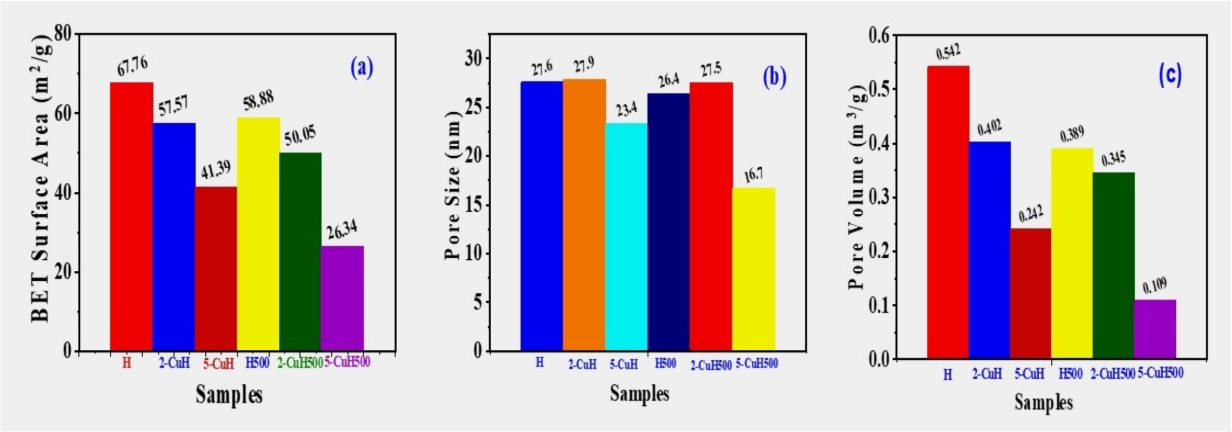

Figure S1. Graphical representation of the BET surface area (**a**), pore diameter (**b**), and pore volume (**c**) of the six synthesized material samples.

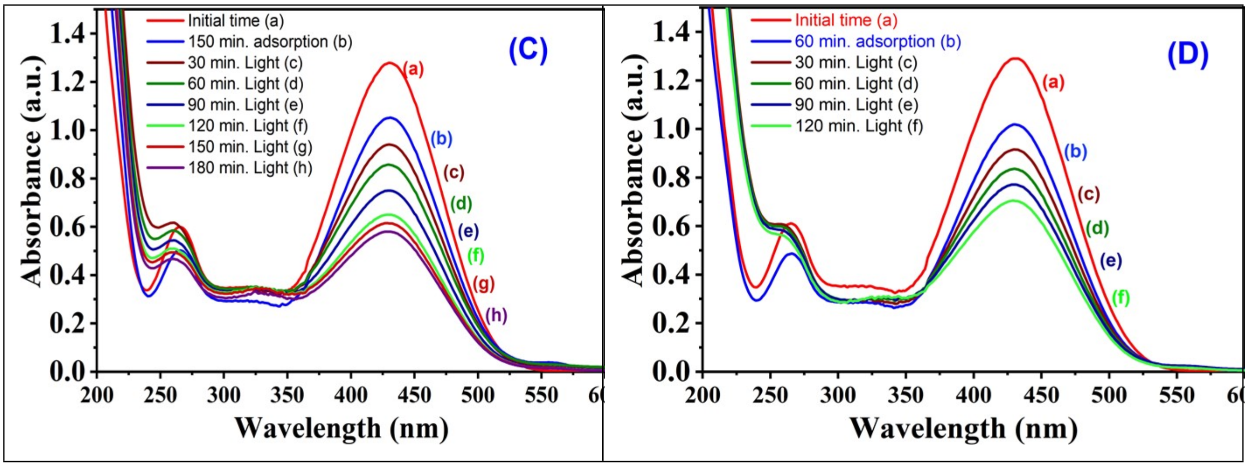

Figure S2. UV–Vis spectra showing the temporal evolution of PR concentration in the presence of isopropyl alcohol using 6-CuH (C) and 5-CuH500 (D), respectively.

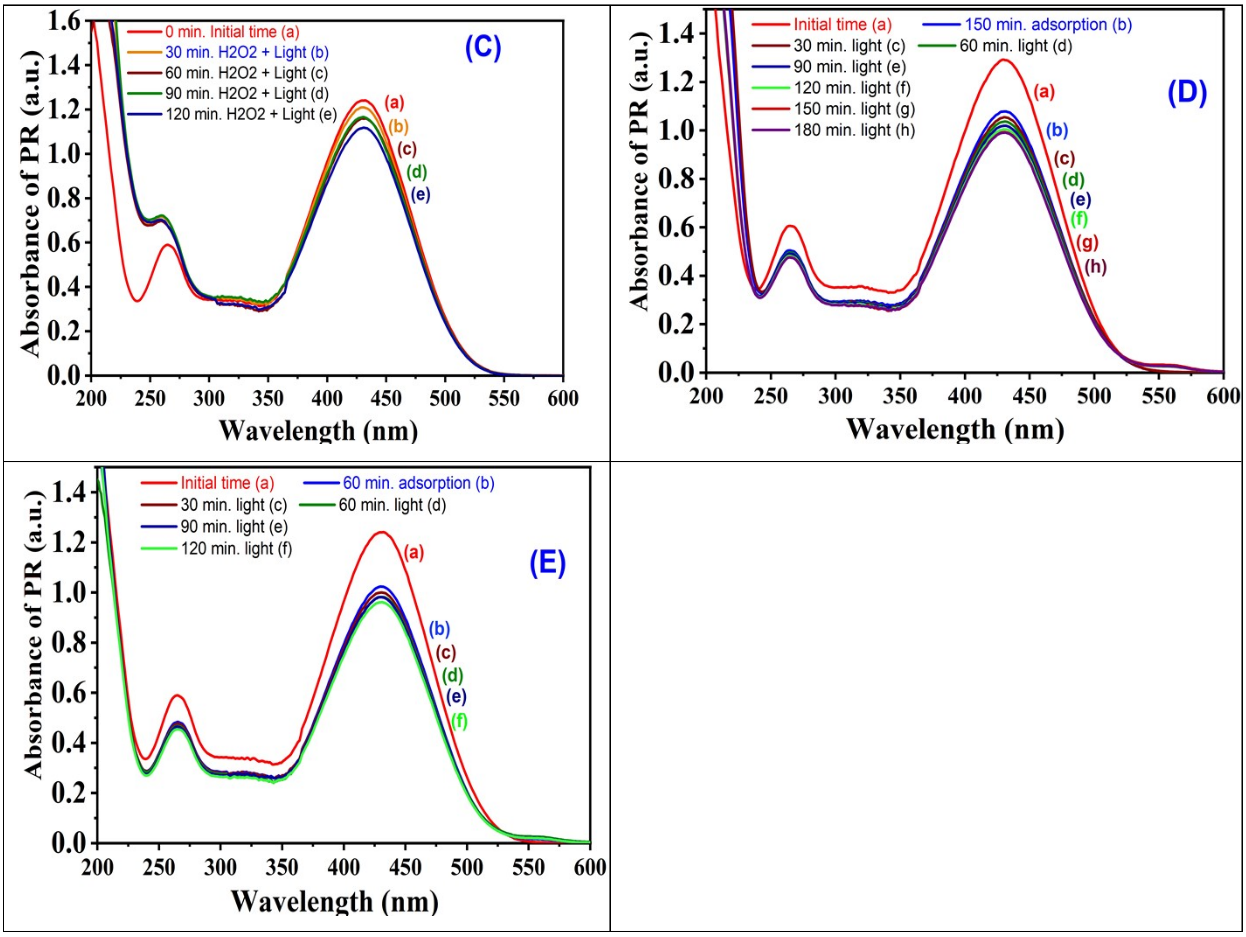

Figure S3. UV-Vis absorption spectra of PR solution in the presence of 30% H_2O_2 and light at pH 3.0 (without catalyst) (C); UV-Vis spectra with only 6-CuH and light (D); UV-Vis spectra with only 5-CuH500 and light (E).

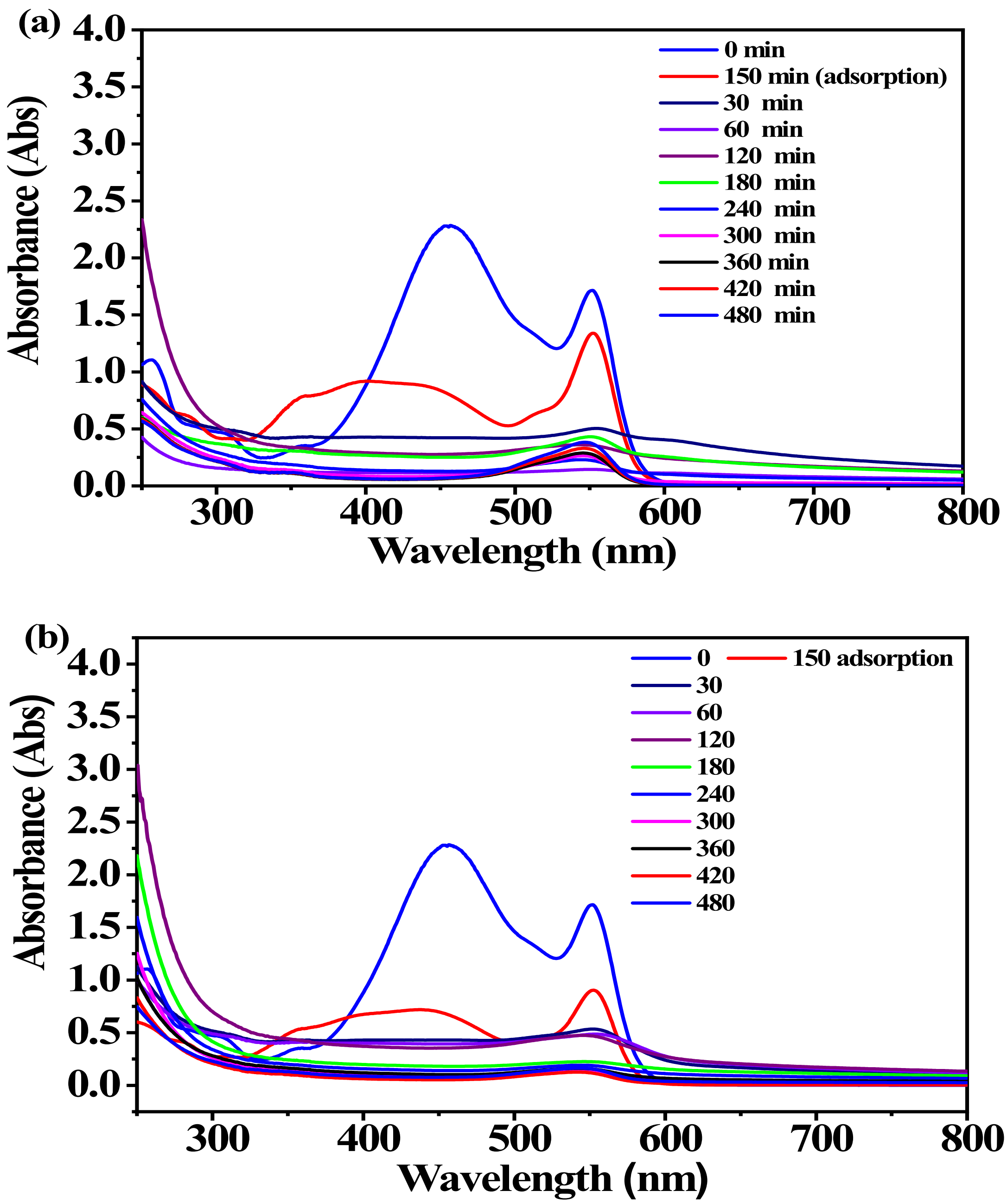

Figure S4. Time-dependent UV-Vis spectra of dyes in mat-dyeing wastewater treated with (**a**) 5-CuH (pH 6.5), and (**b**) 5-CuH500 (pH 4.07)
